# Diversity and Composition of Sulfate-Reducing Microbial Communities Based on Genomic DNA and RNA Transcription in Production Water of High Temperature and Corrosive Oil Reservoir

**DOI:** 10.3389/fmicb.2017.01011

**Published:** 2017-06-07

**Authors:** Xiao-Xiao Li, Jin-Feng Liu, Lei Zhou, Serge M. Mbadinga, Shi-Zhong Yang, Ji-Dong Gu, Bo-Zhong Mu

**Affiliations:** ^1^State Key Laboratory of Bioreactor Engineering and Institute of Applied Chemistry, East China University of Science and TechnologyShanghai, China; ^2^Shanghai Collaborative Innovation Center for Biomanufacturing TechnologyShanghai, China; ^3^School of Biological Sciences, The University of Hong KongHong Kong, Hong Kong

**Keywords:** subsurface petroleum reservoirs, sulfate-reducing microorganisms (SRM), *aprA*, *dsrA*, RT-qPCR

## Abstract

Deep subsurface petroleum reservoir ecosystems harbor a high diversity of microorganisms, and microbial influenced corrosion is a major problem for the petroleum industry. Here, we used high-throughput sequencing to explore the microbial communities based on genomic 16S rDNA and metabolically active 16S rRNA analyses of production water samples with different extents of corrosion from a high-temperature oil reservoir. Results showed that *Desulfotignum* and *Roseovarius* were the most abundant genera in both genomic and active bacterial communities of all the samples. Both genomic and active archaeal communities were mainly composed of *Archaeoglobus* and *Methanolobus*. Within both bacteria and archaea, the active and genomic communities were compositionally distinct from one another across the different oil wells (bacteria *p* = 0.002; archaea *p* = 0.01). In addition, the sulfate-reducing microorganisms (SRMs) were specifically assessed by Sanger sequencing of functional genes *aprA* and *dsrA* encoding the enzymes adenosine-5′-phosphosulfate reductase and dissimilatory sulfite reductase, respectively. Functional gene analysis indicated that potentially active *Archaeoglobus*, *Desulfotignum*, *Desulfovibrio*, and *Thermodesulforhabdus* were frequently detected, with *Archaeoglobus* as the most abundant and active sulfate-reducing group. Canonical correspondence analysis revealed that the SRM communities in petroleum reservoir system were closely related to pH of the production water and sulfate concentration. This study highlights the importance of distinguishing the metabolically active microorganisms from the genomic community and extends our knowledge on the active SRM communities in corrosive petroleum reservoirs.

## Introduction

Metal corrosion is a major problem for oil production systems, leading to serious economic and safety as well as human health problems (Koch et al., 2002; Guan et al., 2014). It is well-recognized that the majority of corrosion of oil pipelines is associated with microorganisms (Almahamedh et al., 2011). Microbial groups of sulfate-reducing microorganism (SRM), acid-producing fermentative microorganisms, metal-reducers and methanogens are frequently detected in oil transporting pipeline systems (Duncan et al., 2009; Stevenson et al., 2011; Liang et al., 2014). The presence of hydrocarbons and their degradation intermediates, such as volatile fatty acids, as electron donors and carbon sources, and the supply of sulfate from injection water as electron acceptors promote the proliferation of microorganisms in anoxic subsurface oil reservoirs and related oil drilling equipment and transport pipeline system (Biderre-Petit et al., 2011; Vigneron et al., 2016).

Bastin et al. (1926) first reported the presence of sulfate-reducing bacteria in oil field waters. Sulfate reduction can have a deleterious impact on crude oil quality by reservoir souring and biocorrosion of the oil infrastructures (Lewandowski and Beyenal, 2008). The SRM, encompassing members affiliated with four bacterial [Proteobacteria (class Deltaproteobacteria), Nitrospira, Firmicutes, Thermodesulfobacteria] and two archaeal phyla (Euryarchaeota, Crenarchaeota) are commonly considered to be main culprits of MIC under anaerobic conditions (Beech and Sunner, 2004; Enning and Garrelfs, 2013; Müller et al., 2014). SRM contribute to chemical microbial influenced corrosion (CMIC) by reducing sulfate to sulfide using organic electron donors such as organic acids (Dinh et al., 2004; Venzlaff et al., 2012). The produced sulfide can then react with carbon steel to form FeS, resulting in dissolution of metal iron and formation of hydrogen which is then also used by SRM as an electron donor (Voordouw et al., 2016). Recent studies showed that some SRM were capable of iron corrosion via electron extraction from Fe^0^ and the process is referred to as electrical microbial influenced corrosion (EMIC) (Enning et al., 2012; Enning and Garrelfs, 2013). Other microorganisms, such as metal-reducers, acid-producing fermentative microorganisms and methanogens may also contribute to corrosion through different biochemical processes (Zhang et al., 2003; Enning and Garrelfs, 2013; Kip and Veen, 2014; Usher et al., 2014). The acid-producing fermentative organisms and methanogens might indirectly increase corrosion through the production of organic acids or syntrophy with corrosion promoting microorganisms (Kip and Veen, 2014). Studies have found high numbers of methanogens associated with pitting corrosion of steel pipes (Uchiyama et al., 2010; Park et al., 2011). Furthermore, the anaerobic biodegradation of labile fuel components coupled with sulfate respiration greatly contributed to the biocorrosion of carbon steel (Liang et al., 2016). Moreover, it has been reported that thiosulfate-utilizing, sulfide-producing fermentative bacteria such as *Anaerobaculum* sp. were implicated with the biocorrosion of a high-temperature petroleum facility (Liang et al., 2014).

Recently, multiplex *dsrA* and *dsrB* amplicon sequencing approach by using new primers and mock community-optimized bioinformatics have been demonstrated to be adequate for monitoring the spatial distribution and temporal abundance dynamics of sulfite- and SRMs (Pelikan et al., 2015). The molecular approach to track SRM is primarily based on functional genes encoding the key selective enzymes including adenosine-5′-phosphosulfate (APS) reductase (Apr) (Meyer and Kuever, 2007) and dissimilatory sulfite reductase (Dsr) (Muyzer and Stams, 2008). The application of these two different genes (*aprA* and *dsrA*) is complementary for the characterization of sulfate-reducing communities and have been studied in different environmental conditions, such as marine sediments (Blazejak and Schippers, 2011), mangrove sediments (Varon-Lopez et al., 2014), and hydrothermal vents (Frank et al., 2013). The composition and activity of microbial communities may depend on a range of factors, including temperature, and availability of electron donors and acceptors (Duncan et al., 2009; Davidova et al., 2012). Accurate and more specific detection and characterization of microbial communities associated with oil souring and pipeline corrosion are crucial to the development of management strategies to minimize or prevent biocorrosion.

In this study, the diversity and composition of microbial community in production water of Jiangsu oil reservoir were explored by analysis of both 16S rDNA and 16S rRNA. In addition, potentially active SRM were also investigated by using the *aprA* and *dsrA* gene transcripts to gain insights for SRM. Three blocks (six different oil wells) with known reservoir souring and pipeline corrosion history were selected in this investigation for a better understanding of microbial communities and SRM in a high temperature and corrosive petroleum reservoir.

## Materials and Methods

### Sampling and Chemical Analysis

Production water samples were obtained on 19 November 2015 from three different blocks (six wells) of Jiangsu oilfield (Yangzhou, Jiangsu, China), including Block W2 (2-53, 2-71), W9 (9-18, 9-14) and W11 (11-7, 11-6). The depths of these wells ranged from 1499 to 2143 m below ground surface, with temperature from 66.2 to 79.6°C. Blocks W2 (2-53, 2-71) and W11 (11-7, 11-6) oil wells have been water-flooded for about 18 and 10 years, respectively. At W9 block, 9-14 has been water-flooded for about 15 years, while 9-18 has never been water-flooded. Corrosion rates of the wells are shown in **Table 1**.

**Table 1:** Physicochemical properties of the samples from Jiangsu oil reservoirs.

	Block W2		Block W9		Block W11	
			
	2-53	2-71	9-18	9-14	11-7	11-6
Depth (m)	1538	1499	1809	1837	2143	2057
Temperature (°C)	67.6	66.2	79.6	75.8	72.3	71.5
pH	8.12	8.23	8.03	7.82	7.90	7.84
Water flooding (year)	18	18	0	15	10	10
Corrosion rate (mm year^-1^)	0.066	0.064	0.031	0.039	0.101	0.236
TDS (g l^-1^)	23.01	24.49	24.41	25.04	25.4	24.45
Water content (%)	79.3	89.4	96.1	79.4	75.4	94.5
Na^+^ (g l^-1^)	4.14	4.06	5.12	5.68	5.54	3.36
Mg^2+^ (mg l^-1^)	11.54	8.86	18.23	23.31	28.72	16.53
Ca^2+^ (mg l^-1^)	28.17	33.19	36.12	78.65	101.92	63.92
NH_4_^+^ (mg l^-1^)	64.8	24.82	56.84	69.83	58.96	60.18
K^+^ (mg l^-1^)	200.07	24.51	175.13	227.82	215.89	215.29
${SO}_{4}^{2-}$ (mg l^-1^)	244.0	83.4	182.2	297.0	533.7	193.4
${NO}_{3}^{-}$ (mg l^-1^)	1.50	9.11	2.22	1.97	5.97	0.81
${CO}_{3}^{2-}$ (g l^-1^)	3.12	2.95	1.90	1.49	1.10	1.16
S^2-^ (mg l^-1^)	9.39	8.42	9.69	9.87	8.37	6.86
${SO}_{3}^{2-}$ (mg l^-1^)	nd	nd	nd	10.63	nd	nd
Cl^-^ (g l^-1^)	4.03	4.37	6.82	8.23	8.22	4.75
${S_{2}O}_{3}^{2-}$ (mg l^-1^)	92.14	89.65	149.6	126.8	38.54	4.11
Formate (mg l^-1^)	2.36	2.16	12.88	22.08	18.71	0.76
Acetate (mg l^-1^)	48.75	24.48	84.73	108.6	159.26	46.96
Propionate (mg l^-1^)	13.36	5.79	7.92	8.49	12.51	3.98
Butyrate (mg l^-1^)	0.73	0.92	1.12	1.06	1.19	0.86


*nd, not detected; TDS, total dissolved salinity.*

Twenty liters of the production water from each production oil well were collected directly from the production valve at the well head into sterile bottle. Production water of each sample for RNA analysis was filtered through 0.1-μm-pore-size polycarbonate membrane filters (Whatman, United Kingdom). Samples were collected within 30 min and stored with a stop solution (95% ethanol, 5% Trizol) for RNA preservation. All samples were stored on ice and immediately transported back to laboratory for further analysis.

Cations and anions and volatile fatty acids in the water samples were quantified by Ion Chromatography (IC DX-600, Dionex, United States). The detailed description of the method used is available elsewhere (Li C.-Y. et al., 2016). The oil well-characteristics were obtained from Jiangsu Oilfield Company. The corrosion rate (at the oil well-temperature) was determined according to the weight loss method for 1 week. Briefly, carbon steel coupons (20#) were immersed in 250 ml serum bottles filled with each of the well-production water (70 ml) under anaerobic conditions. Serum bottles with 70 ml of filtered production water (by 0.22-μm-pore-size polycarbonate membranes) were used as a control. The bottles were closed with butyl stoppers and aluminum seals after purging with pure N_2_ to remove the O_2_ from the corrosion test. The samples were incubated at oil well-temperature in the dark (Okoro et al., 2014).

### Detection and Enumeration of Bacterial Cells

The number of microorganisms in these samples was quantified after fluorescent staining with 4′-6-diamidino-2-phenylindole (DAPI). 5 ml of production water sample was stained with 25 μL of DAPI (5.0 mg mL^-1^) as described before (Purkamo et al., 2013). Stained samples were concentrated on a black 0.2 μm pore-size polycarbonate membrane filter (Millipore, United States) and rinsed twice with 1 ml of sterilized 0.9% NaCl and examined with an epifluorescence microscope (Olympus BX60, China). The number of cells was enumerated from 30 randomly chosen fields on the filter. The total cell number in the samples was then calculated based on filtering sample volume, the observed area of the filter and total surface area of the filter as described in Nyyssönen et al. (2012).

### DNA, RNA Extraction, and cDNA Synthesis and Sequencing

Total DNA and RNA were extracted from each sample using the genomic DNA Kit (Axygen Biosciences, United States) and High Pure RNA Isolation Kit (Roche, United States) according to the manufacturer’s instructions, respectively. Genomic DNA was removed during the RNA extraction with RNase-Free DNase reagents (TakaRa Bio, Japan). Total extracted RNA was checked for residual genomic DNA by performing a polymerase chain reaction (PCR) using the primers 8F (5′-AGAGTTTGATYMTGGCTCAG-3′) and 805R (5′-GACTACCAGGGTATCTAATCC-3′) to ensure that no amplified product was detectable after running on 1.5% agarose gels (Savage et al., 2010). Total RNA was reverse-transcribed to cDNA using M-MLV reverse transcriptase and random hexamer primers (Sangon Biotech, China). The quality and quantity of cDNA was determined using a NanoDrop2000 spectrophotometer (Thermo Fisher Scientific, United States). All DNA and cDNA were stored at -80°C before PCR amplification and sequencing were performed sequentially.

The bacterial hypervariable regions V4–V5 of the 16S rRNA genes were amplified using the primers 515F (5′-GTGCCAGCMGCCGCGG-3′) and 907R (5′-CCGTCAATTCMTTTRAGTTT-3′) containing a barcode sequences (Ren et al., 2014). Archaeal libraries were built using the primers 344F (5′-ACGGGGYGCAGCAGGCGCGA-3′) and 915R (5′-GTGCTCCCCCGCCAATTCCT-3′) (Ohene-Adjei et al., 2007). PCRs were carried out in triplicate of 25-μl reaction volume using 30–40 ng of template DNA or cDNA. The amplifications were run under the following thermocycling conditions: initial denaturation at 94°C for 3 min; 30 (bacteria) or 42 (archaea) cycles of denaturation at 94°C for 30 s, annealing at 55°C (bacteria) or 58°C (archaea) for 30 s, elongation at 72°C for 30 s, and a final extension at 72°C for 10 min. Triplicate reactions were pooled for each sample prior to purification with AxyPrep DNA Kit (Axygen, United States) and quantification with QuantiFluor^TM^ -ST (Promega, United States). Paired-end sequencing was performed on Illumina Miseq platform (Majorbio, China).

Sequences were quality filtered and processed using the UPARSE (Edgar, 2013) and QIIME software package (Caporaso et al., 2010). Paired-end reads were first merged by using FLASH (minimum overlap 10 bp, maximum mismatch 0.25) (Magoč and Salzberg, 2011). PCR primers were detected and trimmed using Cutadapt (Martin, 2011) allowing for one mismatch. Reads not matching the primers or with read lengths below 200 bp were discarded. Trimmed reads were quality-filtered using USEARCH f*astq_filter* function with a maximum predicted sequences errors rate (maxee) of one (Edgar, 2013). High-quality sequences were aligned against the SILVA reference database and clustered into operational taxonomic units (OTUs) at a 97% similarity levels using uclust (Edgar, 2010). OTUs with only one sequences (singleton) were removed from the dataset for all downstream analyses.

### Construction and Analysis of *aprA* and *dsrA* Gene Transcripts Clone Libraries

Clone libraries of *aprA* and *dsrA* genes were constructed from cDNA samples obtained from the six different oil wells. *aprA* gene was amplified using primer pair aprA-1-FW (5′-TGGCAGATCATGATYMAYGG-3′) and aprA-5-RV′ (5′-GCGCCAACNGGDCCRTA-3′, a slightly modified version of aprA-5-RV) (Aoki et al., 2015) with initial denaturation at 95°C for 2 min followed by 35 cycles of 94°C for 30 s, annealing at 55°C for 30 s, and extension at 72°C for 30 s. Similarly, the *dsrA* gene was amplified with the primers DSR-1Fdeg (5′-ACSCAYTGGAARCACG-3′) and PJdsr853Rdeg (5′-CGGTGMAGYTCRTCCTG-3′) (Quillet et al., 2012). Thermal cycling was performed as follows: initial denaturation at 94°C for 2 min; 30 cycles of denaturation at 94°C for 30 s, annealing at 54°C for 30 s, elongation at 72°C for 30 s, and a final extension at 72°C for 10 min. The PCR products (approximately 400 and 450 bp for *aprA* and *dsrA*, respectively) were confirmed by electrophoresis in a 1% (w/v) agarose gel in 1× TAE, and the expected-size PCR products were purified using gel extraction kit. After purification, the products were cloned into *Escherichia coli* using pMD^®^19-T simple vector kit (Takara Biotechnology, Japan) according to the manufacturer instructions. White clones were picked into 1 ml Luria Broth (LB) medium added with ampicillin and incubated at 37°C for 24 h. Primer set M13-47 (5′-CGCCAGGGTTTTCCCAGTCACGAC-3′) and RV-M (5′-GAGCGGATAACAATTTCACACAGG-3′) was used to determine the positive clones. Sequencing of the positive clones was performed on an ABI 377 automated sequencer. Vector sequences were removed and nucleotide sequences were checked for possible chimeric artifacts by BLASTn alignment analysis (Nilsson et al., 2010). OTUs were classified using the BlastClust tool^1^ with the 97% similarity and one representative sequence from each OTU was selected for phylogenetic tree construction. The OTUs of *aprA* and *dsrA* functional genes were translated into amino acids using EMBOSS Transeq^2^ and aligned using MAFFT version 7 (Katoh and Standley, 2013). A neighbor-joining tree was constructed using MEGA version 6 (Tamura et al., 2013). The resulting trees were displayed using the FigTree version 1.4.2^3^.

### Quantification of 16S rRNA and *dsrA* Gene Transcripts by Real-Time PCR on cDNA

The total number of bacterial 16S rRNA and *dsrA* gene transcripts was determined for each sample of this study. The quantification was performed using a CFX96 thermalcycler (Bio-Rad, United States) with the SYBR Green system. The reactions for the two genes were performed using 25 μl reaction volume with 12.5 μl SYBR Green Master Mix (Takara, Japan), 9.5 μl of ddH_2_O, 0.5 μl of each PCR primers, and 2 μl of cDNA sample. The 16S rRNA gene transcript was amplified using the following primer sets: 338F (5′-ACTCCTACGGGAGGCAG-3′) and 805R (5′-GACTACCAGGGTATCTAATCC-3′) (Yu et al., 2005). The amplification was carried out as follows: an initial denaturation step of 4 min at 94°C, 40 cycles of denaturation at 94°C for 30 s, annealing at 57°C for 30 s and extension at 72°C for 1 min. The *dsrA* gene transcripts were amplified with the same primers (Quillet et al., 2012) as described above for the clone library. The PCR reaction conditions were as follows: initial denaturation at 94°C for 2 min; 40 cycles of denaturation at 94°C for 30 s, annealing at 54°C for 30 s, extension at 72°C for 30 s, and a final extension step at 72°C for 5 min. Annealing temperature was experimentally optimized to maximize the specificity of amplification (data not shown). The specificity of this primer pair was checked using the ProbeCheck^4^ against *dsrAB* database which contains 7,695 publicly available partial (6403) and full length (1,292) sequences (Müller et al., 2014). The standard curves for RT-qPCR were generated through 10-fold serial dilutions of plasmids carrying the specific target gene inserts (each run in triplicate). No-template negative controls were used to check for cross contamination. The size of the PCR product was checked with agarose gel electrophoresis. Melting curve (range: from 65 to 95°C) analysis was also conducted following each assay to confirm the specificity of the primer pairs. Amplification efficiency was calculated based on the respective standard curve. Results were expressed in gene copy numbers per ml of production water.

### Bioinformatics and Statistical Analysis

*aprA* and *dsrA* diversity calculations were performed with the EstimateS (Colwell et al., 2012) including Shannon and reciprocal of Simpson index. Sampling coverage was evaluated by using non-parametric richness estimators ACE (abundance-based coverage estimator) (Chao and Yang, 1993), Chao1 (Chao, 1984; Hughes et al., 2001) and Coverage. Rarefaction curves were generated using GraphPad Prism 6. Bubble plots were made using the software R v3.2.4 (R Core Team, 2013) with the ggplot2 (Wickham, 2009) and reshape2 (Wickham, 2012) package. The Pearson correlation coefficients (r) between the gene copy numbers and environmental variables and Spearman′s rank correlations (r_s_) between the relative abundance of individual taxa and environmental factors were investigated via in R software with *Hmisc* package (Harrell, 2012). Differences in total and active microbial community composition were visualized through Non-metric Multi-Dimensional Scaling (NMDS) using Bray–Curtis similarity index by PAST software (version 3.09) (Hammer et al., 2001). OTU abundances for bacteria and archaea underwent square-root transformation to reduce the stress. Two-way PERMANOVA was performed to tests the differences in bulk and active bacterial and archaeal communities and location of community. The Bray–Curtis dissimilarity was calculated for each bacterial and archaeal genomic and active community based on OTU abundance and analyzed via PERMANOVA (999 permutations) in the PAST. Canonical correspondence analysis (CCA) was performed in CANOCO 4.5 for windows to identify the relationships between sulfate-reducing community structure and environmental parameters (Braak, 1988).

### Nucleotide Sequence Accession Numbers

High-throughput raw sequences data have been deposited in the NCBI BioProject database under accession number SRP075241. The 966 nucleotide sequences of the *aprA* and *dsrA* genes reported in this study were deposited in GenBank under the accession numbers SRP075241 to KX300036.

## Results

### Geochemical Characteristics of Production Water

The production water samples possessed high Na^+^ (3.36–5.68 g l^-1^), Cl^-^ (4.03–8.23 g l^-1^), and ${CO}_{3}^{2-}$ (1.10–3.12 g l^-1^). The ${SO}_{4}^{2-}$ concentrations of the six production water samples ranged from 83.4 (2-71) to 533.7 mg l^-1^ (11-7), and the ${S_{2}O}_{3}^{2-}$ concentrations from 4.11 to 149.6 mg l^-1^. The amount of S^2-^ ranged from 6.86 to 9.87 mg l^-1^. Volatile fatty acids including formate, acetate, propionate, and butyrate were detected in all samples (**Table 1**).

### Total Microbial Cell Numbers and Gene Transcript Copy Numbers

**FIGURE 1:**
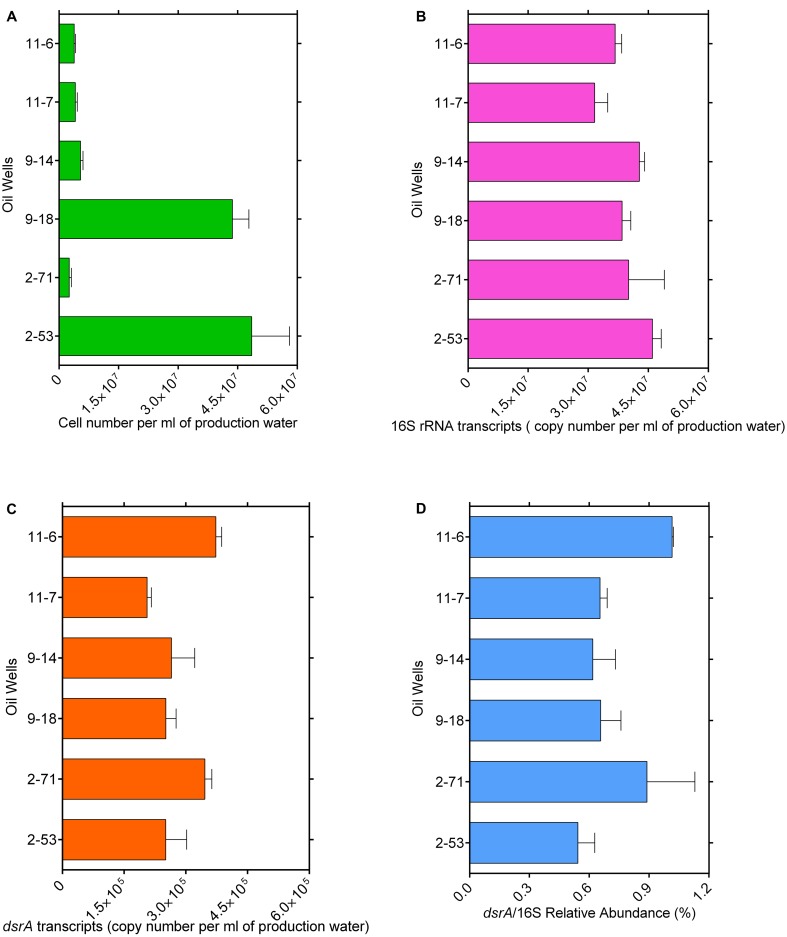
Total microbial cell numbers **(A),** gene transcript levels of bacterial 16S rRNA **(B)** and *dsrA* **(C)** from each well with different corrosion rate, ratios of *dsrA*/16S rRNA copy number **(D)**. Error bars represent standard errors of the means across three replications.

Bacterial cell numbers were high in samples 2-53 (4.8 × 10^7^ cells ml^-1^) and 9-18 (4.3 × 10^7^ cells ml^-1^) and they ranged from 2.5 × 10^6^ to 5.4 × 10^6^ cells ml^-1^ in other samples (**Figure 1A**). The gene transcript levels of bacterial 16S rRNA and *dsrA* were assessed with quantitative PCR. DSR-1Fdeg is either a perfect match or contains only a single mismatch and is likely to amplify to 6228/7695 = 81% of sequences, PJdsr853Rdeg matches perfectly with almost all sequences in the Müller database and the coverage was 92%. Therefore, the exclusion of some sequences by these primers could result in a slight underestimate of *dsrA* transcript abundance. The efficiency values for 16S rRNA and *dsrA* gene transcripts were 93.7 and 80.5% with *R*^2^ values of 0.996 and 0.993, respectively. The abundance ranged from 1.46 × 10^7^ to 3.85 × 10^7^ copies ml^-1^ for 16S rRNA gene transcripts and from 2.06 × 10^5^ to 3.73 × 10^5^ copies ml^-1^ for *dsrA* gene transcripts (**Figures 1B,C**). The ratios of *dsrA* to the 16S rRNA gene transcripts copy numbers were used to determine the relative abundances of active SRMs in the whole community, and the ratios ranged from 0.54 to 1.02% in these samples (**Figure 1D**). The correlation analyses showed that the log-transformed *dsrA* gene transcripts were negatively correlated with the concentration of acetate and propionate significantly (*r* < -0.7, *p* ≤ 0.05). The *dsrA*/16S rRNA ratios were negatively correlated with the concentration of S^2-^ (*r* = -0.85, *p* = 0.03) and propionate (*r* = -0.86, *p* = 0.03) (Supplementary Table S1).

### Bacterial and Archaeal Sequencing and Richness

Supplementary Table S2 shows the results of sequencing of the genomic community (16S rRNA gene amplicons, DNA) and the active community (16S rRNA amplicons, RNA) in the production water of the six oil wells. Our data set consisted of 28,3246 bacterial reads, which clustered into 5,923 bacterial OTUs. Shannon indexes were ranged from 2.15 to 5.75 and Simpson indexes between 0.50 and 0.95 in bacterial libraries. The highest DNA diversity was measured in sample 9-18 and the lowest diversity in sample 11-7 with Shannon indexes 5.75 and 3.15, respectively. The highest RNA diversity was found in sample 11-6 (Shannon index: 4.99) and the lowest in sample 9-14 (1.79). Genomic DNA-based bacterial communities exhibited higher diversity than those of RNA-based active bacterial communities (pairwise *t*-test: *t* = 2.39, *p* = 0.037). The archaeal DNA and cDNA dataset had 26,2116 reads, 1,589 archaeal OTUs. The biodiversity (Shannon index) in genomic DNA-based archaeal communities were significantly more diverse than RNA-based active communities (*p* = 0.01). The Chao 1 richness were between 76 and 276, the Simpson indexes were 0.60–0.93 (Supplementary Table S2).

### Bacterial and Archaeal Community Structure

**FIGURE 2:**
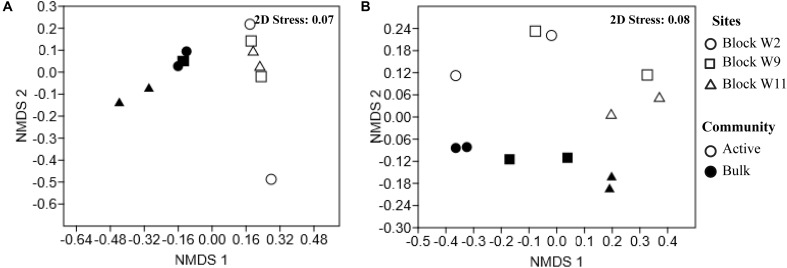
Non-metric Multi-Dimensional Scaling (NMDS) of the Bray–Curtis similarity of the relative abundance of bacterial **(A)** and archaeal **(B)** communities reveal spatial variation by reservoir blocks and between the genomic and active communities. Each point represents an individual sample in the NMDS charts. Full circles represent the genomic communities (DNA-based); empty circles symbol active communities (cDNA-based). Sites are represented by different shapes, block W2 (circle), block W9 (square), block W11 (triangle).

Non-metric Multi-Dimensional Scaling ordination shows a clear separation for both genomic DNA and the active RNA based communities as shown in **Figure 2**. Bacterial community composition differed between the genomic DNA and the active RNA based communities (*F* = 7.9, *p* = 0.002). No significant difference was detected in bacterial community structures between sites across the oil well blocks of this study (*F* = 1.6, *p* = 0.1) (**Figure 2A**). A two-way PERMANOVA analysis also revealed significant differences between the genomic and active archaeal communities (*F* = 4.3, *p* = 0.01) as well as between oil wells across the oil well block (*F* = 4.1, *p* = 0.003) (**Figure 2B**).

### The Genomic and Active Communities and Composition

**FIGURE 3:**
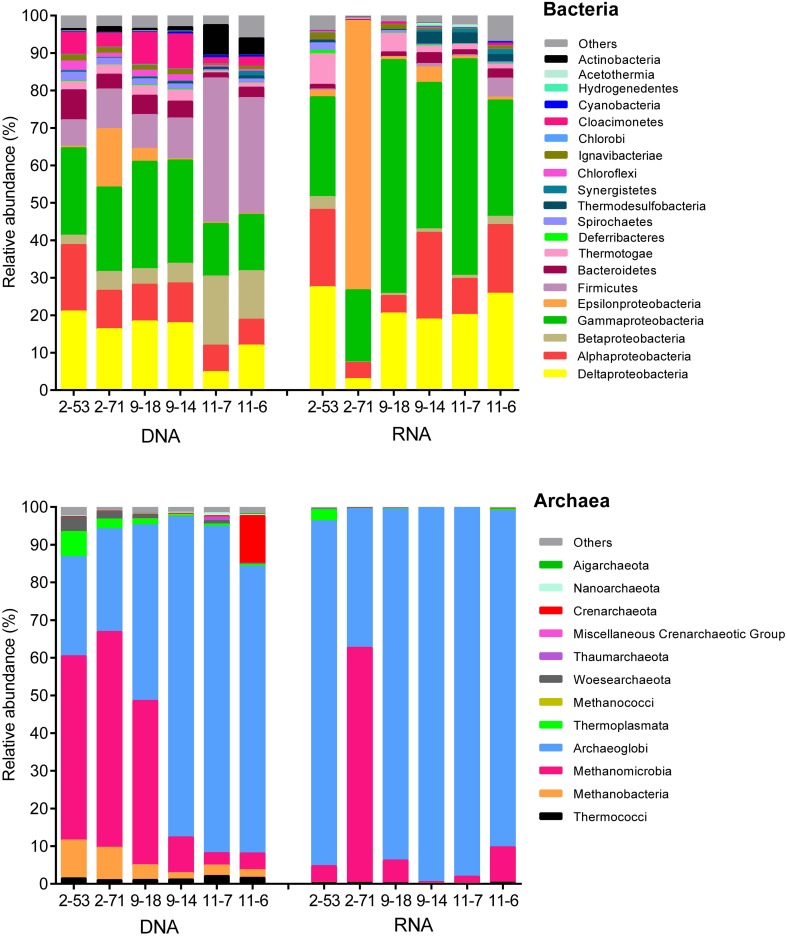
Composition of the genomic (DNA) and active (RNA) communities of production water from six different oil wells. DNA and RNA represent the abundance of rDNA and rRNA-derived 16S sequences of bacteria and archaea.

**Figure 3** shows that the genomic bacterial communities (DNA-based) were dominated by Proteobacteria (44–69% of sequences) and Firmicutes (7.1–38%). Other phyla, such as Bacteroidetes (1.4–8.0%), Deferribacteres (0.050–0.23%), Chloroflexi (0.45–2.6%), Thermotogae (0.51–2.5%), and Spirochaetes (0.29–1.7%) were also represented in the samples. *Desulfotignum*, *Desulfovibrio*, *Desulfarculus*, and *Thermodesulforhabdus* dominated in both the genomic and the active SRM detected in oil reservoirs. *Desulfotomaculum*, *Thermodesulfovibrio*, and *Desulfoglaeba* were detected with low abundance (Supplementary Figure S1). A total of 17 genera of the genomic SRM were inferred from the genomic bacterial community accounting for 19.89, 15.51, 17.47, 17.14, 4.55, and 11.91% of total bacteria in 2-53, 2-71, 9-18, 9-14, 11-7, and 11-6 oil wells (Supplementary Table S3), respectively. The genomic archaeal communities were dominated by Archaeoglobi (26–86%), Methanobacteria (1.7–10%), and Methanomicrobia (3.3–57%). In addition, minor amounts of sequences related to Thermococci (0.97–2.1%), Crenarchaeota (0.070–13%), Woesearchaeota (0.14–3.9%), and Thermoplasmata (0.37–6.7%) were also obtained from the production waters.

The potentially active bacterial communities (RNA-based) were dominated by Proteobacteria, which represented 78–98% of the total sequences, followed by Firmicutes (0.050–5.1%), Bacteroidetes (0.23–2.9%), and Thermotogae (0.34–8.2%) (**Figure 3**). The 16S rRNA bacterial (RNA-based) sequencing also resulted in 17 genera of active SRM. Those SRM occupied 23.90, 2.99, 19.97, 18.57, 20.62, and 25.53% in 2-53, 2-71, 9-18, 9-14, 11-7, and 11-6 oil wells, respectively (Supplementary Table S5). Active archaeal communities were dominated by Archaeoglobi (37–99%) and Methanomicrobia (0.31–62%) as shown in **Figure 3**.

### Comparison of Relative Abundances in the Genomic and Active Communities

**FIGURE 4:**
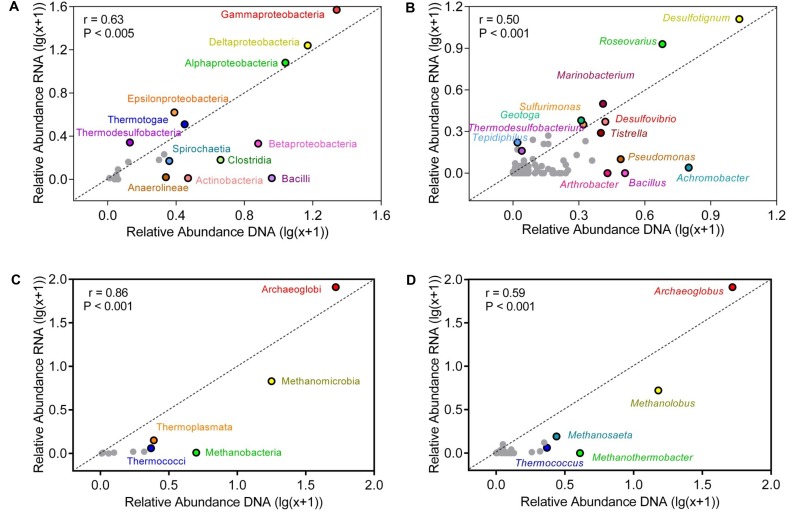
Correlation of relative abundances of taxa in bulk and active microbial communities at class **(A,C)** and genus **(B,D)** level. **(A,B)** represent bacterial communities **(C,D)** represent archaeal communities. The abundance shown was averaged across all samples. Relative abundances were log-transformed to reduce skewness of the data. Points above the diagonal indicated those taxa enriched in the RNA amplicon libraries. Gray points indicate taxa, which occurred in low numbers.

At bacterial class level, Gammaproteobacteria, Deltaproteobacteria, and Alphaproteobacteria dominated the production water communities (**Figure 4**). The relative abundances of class in the active and the genomic bacterial communities were correlated (Spearman correlation: *r* = 0.63, *p* < 0.005), Gammaproteobacteria were more represented in the RNA amplicon libraries compared with the genomic DNA amplicon libraries (pairwise *t*-test: *t* = 2.42, *p* = 0.035), while Betaproteobacteria were more represented in the genomic DNA amplicon libraries (*t* = 3.82, *p* = 0.003). Bacilli (*t* = 5.54, *p* < 0.001), Clostridia (*t* = 4.63, *p* < 0.001), Anaerolineae (*t* = 4.96, *p* < 0.001) were less abundant in the RNA amplicon libraries compared to the genomic DNA amplicon libraries. At genus level, the correlation of relative abundances between the genomic and the active communities was weaker (Spearman correlation: *r* = 0.50, *p* < 0.005) (**Figures 4B,D**). *Desulfotignum* and *Roseovarius* were highly abundant in both the genomic and the active communities. *Thermodesulfobacterium* (*t* = 2.27, *p* = 0.046) and *Tepidiphilus* (*t* = 2.39, *p* = 0.038) were more abundant in RNA amplicon libraries, while *Pseudomonas* (*t* = 4.84, *p* < 0.001), *Alcanivorax* (*t* = 2.59, *p* = 0.027), *Achromobacter* (*t* = 6.74, *p* < 0.001), *Bacillus* (*t* = 3.74, *p* = 0.0038), and *Arthrobacter* (*t* = 3.37, *p* = 0.0071) were more abundant in the genomic DNA amplicon libraries than the RNA amplicon libraries.

Compared with the bacterial communities, the correlation of relative abundances between genomic and active archaeal communities was stronger (Spearman correlation: *r* = 0.86, *p* < 0.001) (**Figure 4**). Thermococci (*t* = 9.25, *p* < 0.001) and Methanobacteria (*t* = 6.54, *p* < 0.001) were more represented in the genomic DNA amplicon libraries compared with the RNA amplicon libraries. Relative abundances of all detected archaeal genera are shown in Supplementary Table S3. *Archaeoglobus* and *Methanolobus* dominated the archaeal community at genus level, and the correlation of relative abundances between the genomic and the active communities was weak (*r* = 0.59, *p* < 0.001). *Thermococcus* (*t* = 9.24, *p* < 0.001) and *Methanothermobacter* (*t* = 4.83, *p* < 0.001) were more abundant in genomic DNA amplicon libraries than RNA amplicon libraries.

### Relationships between Environmental Variables and Microbial Taxa

Correlations between taxa and geochemical data provide insight into the relationship between microbial taxa and environmental factors. Supplementary Table S4 shows that Gammaproteobacteria, Bacteroidia, Chlorobia, and Thermotogae of the genomic DNA were positively correlated with S^2-^ and ${S_{2}O}_{3}^{2-}$ (*r*_s_ > 0.75) and negatively correlated with corrosion rate (*r*_s_ < -0.75). Thermodesulfobacteria were negatively correlated with pH and ${CO}_{3}^{2-}$ (*r*_s_ < -0.80) and positively correlated with oil reservoirs depth (*r*_s_ = 0.94). Deferribacteres were positively correlated with S^2-^ (*r*_s_ = 0.88), Betaproteobacteria were positively correlated with TDS and Ca^2+^ (*r*_s_ > 0.75) and negatively correlated with ${CO}_{3}^{2-}$ (*r*_s_ = -0.94). Methanobacteria, Thermoplasmata, and Woesearchaeota were positively correlated with pH (*r*_s_ > 0.75). For genomic DNA, Archaeoglobi were positively correlated with oil reservoirs depth, TDS, Mg^2+^, Ca^2+^, and Cl^-^ and negatively correlated with ${CO}_{3}^{2-}$ (*r*_s_ = 0.89). Methanococci were negatively correlated with corrosion rate (*r*_s_ = -0.88), whereas Crenarchaeota were positively correlated with corrosion rate (*r*_s_ = 0.90).

Supplementary Table S5 shows the active Betaproteobacteria and Deferribacteres were negatively correlated with ${NO}_{3}^{-}$ (*r*_s_ < -0.75). Active Thermodesulfobacteria were positively correlated with ${SO}_{4}^{2-}$ (*r*_s_ = 0.89) and negatively correlated with pH (*r*_s_ = -0.89). Thermococci, Methanomicrobia, and Crenarchaeota were negatively correlated with formate and acetate (*r*_s_ < -0.80). Active Archaeoglobi were positively correlated with Mg^2+^, ${SO}_{4}^{2-}$, Na^+^, formate, and acetate (*r*_s_ > 0.80).

### Sequence Analysis of *aprA* Gene

**FIGURE 5:**
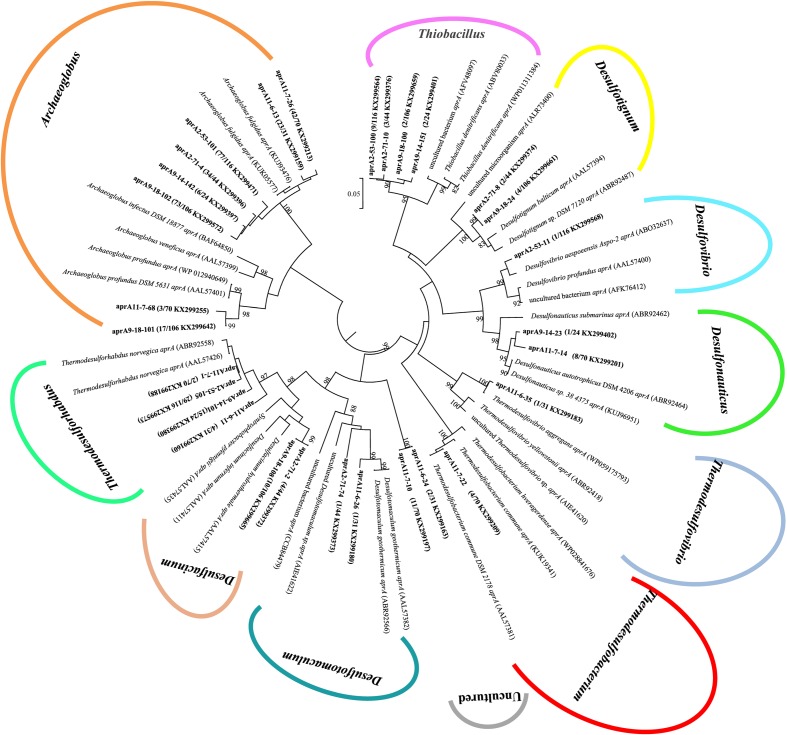
Phylogenetic tree of deduced amino acid sequences of *aprA* gene amplicons from six different oil wells of production water. The topology of the tree was obtained by the neighbor-joining method. Bold black letters indicated the OTUs detected in this study. The OTUs are shown with clone names, the number of clones of each OTU and accession numbers. The bootstrap values at the nodes of >80% (n = 1000 replicates) are reported. The scale bar represents 5% sequence divergence.

A total of 391 *aprA* gene clones were clustered into 29 OTUs based on sequence similarity at 97% threshold value. The rarefaction curves for samples 9-18 and 11-7 *aprA* gene clone libraries reached saturation (100% coverage) and others did not plateau with coverage values ranged from 93.5 to 99.1% (Supplementary Figure S2A and **Table 2**). *aprA* sequences related to known SRM comprised 92.5% of total *aprA* gene sequences (23 OTUs), only four OTUs (4.1% of all *aprA* gene sequences) corresponded to sulfur-oxidizing microorganisms (SOM), and they all fell within cluster genus *Thiobacillus*. The remaining 13 clones, 2 OTUs (3.3%) without close affiliation to either SOM or SRM lineage, were grouped into aprA11-6-24 and aprA11-7-10, only found in Block W11 as shown in **Figure 5**.

**Table 2:** Biodiversity and predicted richness of the *aprA* and *dsrA* gene sequences.

	Samples	No. of sequences	OTUs	Coverage	Shannon diversity index	Simpson (inverse) index	S_chao1_	S_ACE_
*aprA*	2-53	116	4	0.991	0.86	1.96	4	5.09
	2-71	44	5	0.977	0.83	1.63	5	5.44
	9-18	106	5	1	0.97	1.96	5	5
	9-14	24	4	0.958	0.98	2.17	4	4.94
	11-7	70	6	1	1.25	2.48	6	6
	11-6	31	5	0.935	0.88	1.74	5.48	7.22

*dsrA*	2-53	77	4	1	1.17	2.77	4	4
	2-71	50	3	1	0.57	1.44	3	3
	9-18	114	4	1	0.71	1.53	4	4
	9-14	86	2	1	0.5	1.46	2	2
	11-7	136	5	0.985	0.56	1.39	5.99	8.67
	11-6	112	2	1	0.59	1.67	2	2


**FIGURE 6:**
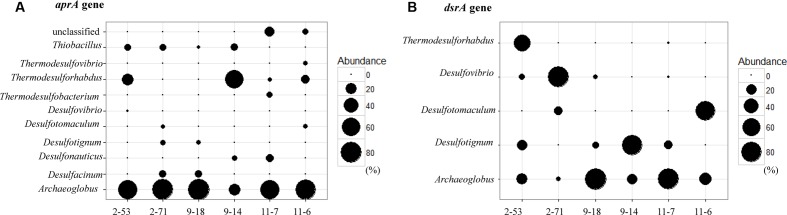
Bubble plots of *aprA* **(A)** and *dsrA* **(B)** gene community structures showing the relative abundances across samples. The size of each bubble indicate the relative abundance (percentage) of identified *aprA* and *dsrA* gene sequences falling within taxonomic group (at the genus level).

At the genus level, the relative abundance of *Archaeoglobus*-affiliated *aprA* genes comprised 66, 77, 85, 25, 64, and 74% of the clone libraries from the production water of 2-53, 2-71, 9-18, 9-14, 11-7, and 11-6, respectively (**Figure 6A**). *Desulfotignum*-affiliated *aprA* genes were detected in 2-71 (4.5%) and 9-18 (3.8%) samples. aprA2-53-11 and aprA2-53-105 which belonged to the genus *Desulfovibrio* and *Thermodesulforhabdus* were detected in sample 2-53. In addition, sequences related to *Desulfacinum* (9.1%) and *Desulfotomaculum* (2.3%) were detected in 2-71. *Desulfonauticus*, *Thermodesulfobacterium*, and *Thermodesulforhabdus* were also found in 11-7 with a relatively low frequency, resulting in 11, 5.7, and 2.9% of all clones, respectively. Sequences related to *Desulfotignum* and *Desulfacinum* were less abundant, but were detected at 9-18, accounting for about 3.8 and 9.4% of the clones, respectively. aprA9-14-101 and aprA9-14-23 related to *Thermodesulforhabdus* and *Desulfonauticus* were detected in sample 9-14, which represented 63 and 4.2% of total *aprA* gene sequences, respectively.

### Sequence Analysis of *dsrA* Genes

**FIGURE 7:**
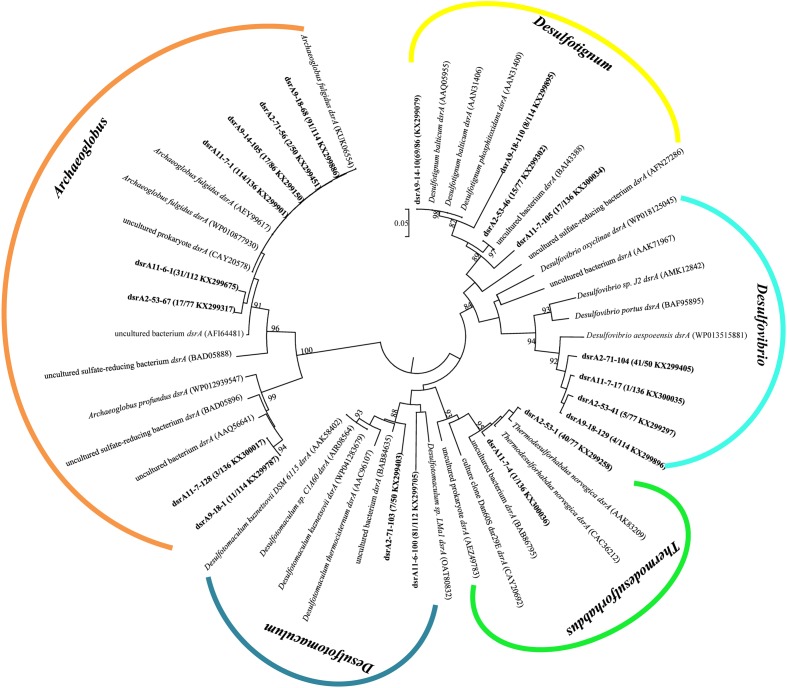
Phylogenetic tree of deduced amino acid sequences of *dsrA* gene of production water from six different oil wells. The topology of the tree was obtained by the neighbor-joining method. Bold black letters indicated the OTUs detected in this study. The OTUs are shown with clone names, the number of clones of each OTU and accession numbers. The bootstrap values at the nodes of >80% (*n* = 1000 replicates) are reported. The scale bar represents 5% sequence divergence.

A total of 575 *dsrA* gene clones were grouped into 20 OTUs, and the rarefaction curves for *dsrA* gene clone libraries reached clear saturation except for W11-7 (98.5% coverage) (Supplementary Figure S2B). Richness and diversity as supported by Shannon index and Chao 1 values were low in samples from different wells (**Table 2**). The majority of *dsrA* sequences were affiliated to those of *Archaeoglobus* (286 sequences, 8 OTUs). 109 sequences (4 OTUs) were related to *Desulfotignum* and 51 sequences (3 OTUs) clustered with *Desulfovibrio*. 41 sequences (2 OTUs) were related to *Thermodesulforhabdus* and 88 sequences (2 OTUs) belonged to *Desulfotomaculum* as shown in **Figure 7**.

dsrA9-18-1 and dsrA11-7-128 were 95% identical to *Archaeoglobus profundus*, other OTUs belonging to *Archaeoglobus* were 97–99% identical to *Archaeoglobus fulgidus*, which is known to reduce both sulfate and thiosulfate (Lenhart et al., 2014). Sequences related to *Archaeoglobus* were detected in all samples and also identified as the major component in 9-18 and 11-7 with 89 and 86% of the clone library, respectively. Sequences affiliated to *Desulfotignum* were detected in 9-18, 9-14, 2-53, and 11-7 (**Figure 6B**). Their closest relative was *Desulfotignum balticum*, and *Desulfotignum* group was dominant in 9-14 (80% of *dsrA* clones in library). Four samples (2-53, 2-71, 9-18, and 11-7) were detected for sequences affiliated to *Desulfovibrio*, with the highest abundance (82% of the *dsrA* gene clone library) detected in 2-71. In contrast, in 2-53, *Thermodesulforhabdus* was the most abundant (52% of all clones), which was also detected in W11-7 (0.74% of the clone library). The genus *Desulfotomaculum* comprised of 14 and 72% of the *dsrA* gene clone libraries obtained from 2-71 and 11-6, respectively (**Figure 6B**).

### Relationships of Sulfate-Reducing Communities with Environmental Parameters

**FIGURE 8:**
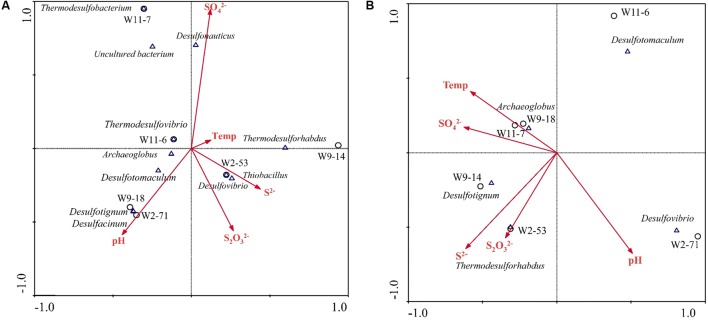
Canonical correspondence analysis (CCA) for the relationships between community structure based on *aprA* **(A)** and *dsrA* **(B)** genes and environmental factors in the six samples. Arrows indicate the direction and magnitude of measurable variables associated with sulfate-reducing community structures. Each circle represents sampling sites.

Based on variance inflation factors with 999 Monte Carlo permutations, five environmental variables, temperature, pH, concentration of S^2-^, ${S_{2}O}_{3}^{2-}$, and ${SO}_{4}^{2-}$ were selected in the CCA biplot. The length of an environmental factors arrow in the ordination plot indicates the strength of the relationship of that parameter to community composition. In the *aprA* gene CCA plot (**Figure 8A**), concentration of ${SO}_{4}^{2-}$ appeared to be the most important environmental parameter, and the genus *Desulfonauticus* was closely associated with the ${SO}_{4}^{2-}$. *Desulfovibrio* were associated with ${S_{2}O}_{3}^{2-}$ and S^2-^ levels. The genera *Desulfotignum* and *Desulfacinum* were correlated with pH. The relationship of *dsrA* gene-based community with environmental factors (**Figure 8B**) showed that *Archaeoglobus* were positively correlated to temperature and *Thermodesulforhabdus* were associated with both ${S_{2}O}_{3}^{2-}$ and S^2-^ levels.

## Discussion

Oil reservoirs are generally characterized with a high content of hydrocarbons and anoxic conditions. Previous studies of oil reservoirs reported that this unique ecosystem harbored diverse and variable microbial communities (Pham et al., 2009; Mayumi et al., 2011; Mbadinga et al., 2012; Tang et al., 2012; Wang et al., 2014; Li X.-X. et al., 2016). This study aimed to characterize genomic and active microbial populations, especially sulfate reducer in oil reservoir production water collected from Jiangsu oilfield which is known for reservoir souring and pipeline corrosion, and contains high sulfate (Guan et al., 2014). Results broaden our understanding on the microorganisms contributing to MIC in extreme environment and development of more specific treatment strategies for mitigation of problems with SRM in such environments.

### Comparative Analysis of DNA and RNA-Derived Communities

In this study, DNA- and RNA-based libraries revealed a diverse microbial community in the production water of six different oil wells which suffered reservoir souring and pipeline corrosion. The DNA-derived bacterial and archaeal communities analyzed by the 16S rRNA gene sequences represent the whole communities including groups that are dormant or inactive, spore and dead (Blagodatskaya and Kuzyakov, 2013; Blazewicz et al., 2013). In contrast, the RNA-based community represents the active community (Moeseneder et al., 2005; Angel et al., 2013; Blazewicz et al., 2013) using ribosomal RNA as an indicator of microbial activity has limitations, including non-linear relation between growth rate and cellular rRNA content, as well as ribosomal content in dormant cells that could be high in some bacteria (Blazewicz et al., 2013). However, although this analysis has several weaknesses, comparison of the DNA- and RNA-based libraries provides for more complete characterization of microorganisms and new insights into activity in environmental communities (Frenk et al., 2015; Nazina et al., 2017).

Compared with RNA libraries, a higher species richness in the bacterial and archaeal DNA libraries was observed (Supplementary Table S2), which is consistent with a study in hydrothermal vent (Lanzén et al., 2011). One major difference between DNA and RNA-based bacterial community composition was that Firmicutes were more abundant in DNA samples, while fewer sequences in RNA samples were identified as Firmicutes (**Figure 3**). It has been reported that Firmicutes is an important component of microbial communities in water-injected and pristine oil reservoirs in Russia based on 16S rRNA gene (Frank et al., 2016). In Dagang oilfield, most sequences belonged to members of the phyla Firmicutes and Proteobacteria according to DNA-derived bacterial clone library. While, in RNA-based clone library, Proteobacteria were the most abundant (Nazina et al., 2017), which is consistent with our results. The dominant bacterial genera were *Desulfotignum* and *Roseovarius*, with the relative abundances ranging from 7.0 to 18.1% and 0.8 to 17.7% respectively (Supplementary Table S3). Tian et al. (2017) also found *Desulfotignum* was one of the dominant populations with its relative abundances in the different high temperature petroleum reservoirs ranging from 9.7 to 43.2%. In archaeal community, the most noticeable difference was that Methanobacteria and Thermococci were more abundant in DNA amplicon datasets (**Figure 3**). The majority of the archaeal OTUs were related to the *Archaeoglobus* (26.1–99.4%) and *Methanolobus* (0.09–60.6%) (Supplementary Table S3) in DNA- and RNA-based libraries. In a hyper-temperature Japanese oil well, the major populations were *Thermotoga*, *Thermodesulfobacterium*, and *Archaeoglobus* (Yamane et al., 2011).

### *Archaeoglobus* as Dominant Active Sulfate-Reducer

16S rDNA and rRNA tag sequencing, as well as phylogenetic analyses of *aprA* and *dsrA* gene transcripts showed that *Archaeoglobus* was the dominant and active sulfate-reducer in production water of corrosive petroleum reservoirs in this study. *Archaeoglobus*, with close relatedness to *Archaeoglobus fulgidus* and *Archaeoglobus profundus*, were found in all production water samples. Three *Archaeoglobus* species are known to contain the complete pathway for dissimilatory sulfate reduction: *Archaeoglobus fulgidus*, *Archaeoglobus profundus*, and *Archaeoglobus lithotrophicus* (Speich and Trüper, 1988; Dahl et al., 1990). The genus *Archaeoglobus* may play a major role in sulfate reduction in production waters of high temperature reservoirs because of the high numbers of *aprA* and *dsrA* gene transcript clones related to *Archaeoglobus*. Interestingly, *Archaeoglobus* was also found and dominated the sulfate-reducing communities in the production water of North Sea high-temperature oil reservoir (Gittel et al., 2009).

Sulfate-reducing members of *Desulfotignum*, *Desulfovibrio*, and *Thermodesulforhabdus* genera were also prevalent groups in the investigated samples. *Desulfotignum* related sequences were closely related to *Desulfotignum balticum*, which is known for anaerobic benzoate degradation coupled with sulfate reduction (Habe et al., 2009). *Desulfovibrio* species can use hydrogen, formate, and many other organic compounds to reduce sulfate (Voordouw, 1995). They have been reported to be strongly adapted to environmental stresses, such as heavy metal contamination (Quillet et al., 2012). It has been reported that *Desulfovibrio* species dominated the microbial communities of highly corrosive biofilms of an offshore oil production facility (Vigneron et al., 2016). *Thermodesulforhabdus* were closely related to *Thermodesulforhabdus norvegicus* which was isolated from hot water of North Sea oilfield (Beeder et al., 1995). We also identified sequences related to *Desulfotomaculum*, which can grow under a range of sulfate concentrations, use diverse organic substrates and participate in syntrophic metabolism with methanogens (Imachi et al., 2006; Aüllo et al., 2013). And members of *Desulfotomaculum* are known for their sporulation capability, which is considered to be one of the microbial strategies used to resist unfavorable temperatures or nutrient deprivation conditions (Aüllo et al., 2013). In addition, some representatives of SRM within Deltaproteobacteria were also observed, which were assigned to *Desulfobotulus*, *Desulfatitalea*, *Desulfocella*, *Desulfobulbus*, *Desulfovibrio*, *Desulfofustis*, *Desulfomicrobium*, *Desulfonauticus*, *Desulfarculus*, and *Desulfoglaeba*. Moreover, *Thermodesulfobacterium* (Thermodesulfobacteria) and *Thermodesulfovibrio* (Nitrospira) were also detected with relatively low abundances.

### Strategies for Mitigation of Petroleum Reservoirs Souring and Pipeline Corrosion

Since the role of microorganisms in MIC was acknowledged, different methods have been applied in the oil and gas industries to control or prevent microbial reservoir souring. So far, control measures against souring in the oil industry include removing sulfate from the injection water or inhibition or killing of SRM by continuous amendment of injection water with biocides, and chemical inhibitors including nitrite and nitrate (Rabus et al., 2015). Due to the high investment and operational cost, sulfate removal from the injection water is not feasible (Liebensteiner et al., 2014). Biocides are commonly used to control SRM in the oil industry, but application of biocides is usually limited to above-ground infrastructures to control microbial souring and corrosion, and the use of biocides is generally a concern with the increased resistance to biocides for long-term applications (Xue and Voordouw, 2015). Amendment of injection water with nitrate is the most widely accepted strategy to control microbial reservoir souring due to the effective and the advantages over biocides (Voordouw, 2011). Nitrate injection is effective in controlling souring by (1) the competitive suppression of SRM by stimulate the growth of heterotrophic nitrate-reducing bacteria (HNRB) which are able to outcompete SRM for the same electron donors, such as organic acids; (2) the removal of generated sulfide by sulfide-oxidizing nitrate-reducing bacteria (SONRB); (3) inhibition of the dissimilatory sulfite reductase (*dsr*) by nitrite (the metabolic intermediate of nitrate reduction) (Liebensteiner et al., 2014). In spite of success with nitrate to control or prevent souring, problems exist for ineffectiveness in low-temperature oil reservoirs as a result of emergence of microbial zone (Callbeck et al., 2011). Recently, perchlorate and monofluorophosphate have been demonstrated as effective inhibitors of SRM (Carlson et al., 2015a,b). Perchlorate is effective in laboratory tests and also inhibits the sulfate reduction by *Archaeoglobus* (Engelbrektson et al., 2014). As oil reservoir properties have a dominant effect in determining the results of souring control measures, a better understanding on the microbial community composition and eco-physiology are important for the development of a specific mitigation strategy.

### Dual Role of Sulfur-Oxidizer in Corrosion

Except for SRM, SOM were also detected in these oil wells by 16S rDNA and rRNA tag sequencing. The SOM could catalyze the inorganic compound sulfide to sulfate. *Roseovarius* and *Sulfurimonas* were the dominant SOM in the samples of this study, with the abundance ranged from 1.8 to 17.7% in the cDNA-based bacterial libraries. The abundance of *Sulfurimonas* in the active bacterial community reached as high as 72% in oil well 2-71. In addition, sulfur-oxidizing species of the *Paracoccus*, *Rhodovulum, Sulfuritalea*, *Dechloromonas, Arcobacter*, and *Rhizobium* genera were also detected with low abundances (Supplementary Table S5). Previous studies have pointed out that some SOM like *Sulfurospirillum* spp. could control SRM and their activity was the primary force to control in nitrate injection systems (Hubert and Voordouw, 2007). SONRB may compete with SRM for electron donors and reduce the concentration of sulfide by oxidizing the dissolved sulfide. However, recent study showed that members of *Sulfuricurvum* and *Sulfurovum* of SOM play a potential role in MIC in pipelines subjected to injection of bisulfate (An et al., 2016). Furthermore, it has been reported that the presence of SONRB can promote the formation of greigite, a product of corrosion, and the *Sulfurospirillum* and *Arcobacter* which have the metabolic capacity of sulfide oxidation with nitrate were enriched by the nitrate-amendment rig (connected to a water injection pipeline) suffering from serious corrosion compared with non-amendment control (Drønen et al., 2014). Sulfuric acid may also induce more serious corrosion and play a certain role in MIC. The SOM community should not be ignored in petroleum reservoirs (Tian et al., 2017).

## Conclusion

The combined approach of 16S rDNA and 16S rRNA high-throughput sequencing and *aprA* and *dsrA* clone libraries provided evidence that a diverse microorganisms inhabited in the corrosive and high temperature petroleum reservoir. *Desulfotignum* and *Roseovarius*, *Archaeoglobus* and *Methanolobus* dominated the bacterial and archaeal communities, respectively. The metabolically active microorganisms differed from the genomic community, representing a subset of the taxa presented in the genomic community. Most detected SRM were affiliated to *Archaeoglobus*, *Desulfotignum*, *Desulfovibrio* and *Thermodesulforhabdus*, they were closely related to pH of the production water and sulfate concentration. The most abundant sequence which belonged to SRM was identified as the genus *Archaeoglobus*, indicating that archaea *Archaeoglobus* might play a major role in reservoir souring and pipeline corrosion in high temperature oil reservoirs.

## Author Contributions

B-ZM and J-DG designed the experiments, X-XL conducted the experiments, X-XL and LZ carried out the microbial analysis. J-FL, S-ZY, and SM gave suggestions for the experiments and results analysis. X-XL prepared the manuscript with contribution from all co-authors.

## Conflict of Interest Statement

The authors declare that the research was conducted in the absence of any commercial or financial relationships that could be construed as a potential conflict of interest.

## Footnotes

1 http://toolkit.tuebingen.mpg.de/blastclust

2 http://www.ebi.ac.uk/Tools/st/emboss_transeq/

3 http://tree.bio.ed.ac.uk/software/figtree/

4 http://131.130.66.200/cgi-bin/probecheck/probecheck.pl
