# Supplementary material for: Diversity and Composition of Sulfate-Reducing Microbial Communities Based on Genomic DNA and RNA Transcription in Production Water of High Temperature and Corrosive Oil Reservoir
Source: Front Microbiol. 2017 Jun 7;8:1011. doi: 10.3389/fmicb.2017.01011 (PMC5461352; doi:10.3389/fmicb.2017.01011)
Supplement: Supplementary file 1 [file Data_Sheet_1.PDF]

Supplementary materials

Diversity and Composition of Sulfate-Reducing Microbial Communities Based on Genomic DNA and RNA Transcription in Production Water of High Temperature and Corrosive Oil Reservoirs

Xiao-Xiao Li[1], Jin-Feng Liu[1], Lei Zhou[1], Serge Maurice Mbadinga[1,3], Shi-Zhong Yang[1], Ji-Dong Gu[2], Bo-Zhong Mu[1,3*]

[1]State Key Laboratory of Bioreactor Engineering and Institute of Applied Chemistry, East China University of Science and Technology, Shanghai, China

[2]School of Biological Sciences, The University of Hong Kong, Pokfulam Road, Hong Kong SAR, China

[3]Shanghai Collaborative Innovation Center for Biomanufacturing Technology, Shanghai, China

*Corresponding author: Bo-Zhong Mu

E-mail: bzmu@ecust.edu.cn

State Key Laboratory of Bioreactor Engineering and Institute of Applied Chemistry
East China University of Science and Technology, Shanghai 200237, PR China

Tel: +86-21-64252063, Fax: +86-21-64252485

Table S1 Pearson correlations between 16S rRNA and *dsrA* transcripts copy number and environmental factors[a].

	**16S rRNA**		***dsrA***		***dsrA/*16S rRNA**	
**Environmental variable**	**r**	***p***	**r**	***p***	**r**	***p***
corrosion rate	-0.40	0.44	0.53	0.28	0.74	0.09
pH	0.37	0.47	0.14	0.79	-0.04	0.94
Temp	-0.21	0.69	-0.36	0.49	-0.27	0.60
SO_4^{2-}	-0.56	0.24	-0.78	0.07	-0.45	0.37
S^{2-}	0.52	0.29	-0.58	0.23	**-0.85**	**0.03**
$S_2O_3^{2-}$	0.53	0.27	-0.3	0.56	-0.59	0.21
Na^+	-0.20	0.70	-0.79	0.06	-0.69	0.13
Mg^{2+}	0.63	0.18	-0.64	0.17	-0.31	0.54
Ca^{2+}	-0.67	0.14	-0.39	0.45	-0.03	0.96
NH_4^+	0.09	0.86	-0.46	0.36	-0.50	0.31
K^+	-0.12	0.82	-0.45	0.37	-0.38	0.46
NO_3^-	-0.31	0.55	0.03	0.96	0.19	0.71
CO_3^{2-}	0.70	0.12	0.15	0.78	-0.20	0.70
Cl^-	-0.45	0.37	-0.64	0.17	-0.42	0.41
Mineralization	-0.72	0.11	-0.17	0.75	0.19	0.72
Formate	-0.28	0.59	-0.69	0.13	-0.55	0.26
Acetate	-0.58	0.22	**-0.81**	**0.05**	-0.49	0.32
Propionate	0.07	0.89	**-0.87**	**0.02**	**-0.86**	**0.03**
Butyrate	-0.66	0.15	-0.54	0.27	-0.20	0.69

[a]The gene transcript numbers were log transformed. Significant differences ($P < 0.05$) are highlighted in bold.

Table S2 Diversity characteristics of bacterial and archaeal 16S rDNA and 16S rRNA.

Sample[a]	No. Reads*	No. OTUs	Chao1 richness	Shannon index	Simpson index	Coverage
**2-53D-B**	23355	614	888	5.68	0.94	0.985
**2-71D-B**	23569	446	665	5.15	0.92	0.990
**9-18D-B**	21053	571	880	5.75	0.95	0.985
**9-14D-B**	22531	506	732	5.53	0.94	0.989
**11-7D-B**	27252	388	464	3.53	0.75	0.992
**11-6D-B**	20514	547	665	4.88	0.86	0.989
**2-53R-B**	26639	533	1055	4.64	0.89	0.983
**2-71R-B**	19370	229	312	2.15	0.50	0.994
**9-18R-B**	28811	498	909	3.02	0.64	0.984
**9-14R-B**	16111*	366	567.88	4.24	0.84	0.991
**11-7R-B**	29278	459	938	3.52	0.71	0.986
**11-6R-B**	24763	766	1151	4.99	0.88	0.98
**2-53D-A**	22869	156	230	4.46	0.92	0.996
**2-71D-A**	14520	113	156	4.00	0.91	0.997

**9-18D-A**	24883	131	180	4.12	0.91	0.997
**9-14D-A**	25278	259	276	5.05	0.93	0.997
**11-7D-A**	17728	128	135	3.15	0.73	0.999
**11-6D-A**	29685	156	201	3.22	0.76	0.997
**2-53R-A**	21231	153	226	3.59	0.87	0.996
**2-71R-A**	29370	80	105	3.01	0.83	0.998
**9-18R-A**	21199	124	165	3.32	0.84	0.997
**9-14R-A**	14460*	61	76	1.79	0.60	0.999
**11-7R-A**	25065	110	127	2.04	0.62	0.998
**11-6R-A**	15828	118	167	2.22	0.64	0.997

[a]All samples were rarified to 16111 for bacteria and 14460 sequences for archaea before diversity indices were calculated. Abbreviations: D, DNA; R, RNA; B, Bacteria; A, archaea.

Table S3 Differences in relative abundances of all detected genera in different oil wells by 16S rDNA and 16S rRNA genes.

		DNA						RNA					
Domain	Genus	2-53	2-71	9-18	9-14	11-7	11-6	2-53	2-71	9-18	9-14	11-7	11-6
Bacteria	*Desulfotignum*	15.64	11.81	13.43	13.51	3.32	7.01	16.24	2.65	18.10	11.11	16.63	16.58
Bacteria	*Desulfobotulus*	0.03	0.02	0.05	0.00	0.00	0.03	0.10	0.01	0.09	0.05	0.01	0.01
Bacteria	*Desulfatitalea*	0.01	0.03	0.02	0.01	0.00	0.00	0.15	0.00	0.04	0.03	0.07	0.03
Bacteria	*Desulfocella*	0.02	0.01	0.01	0.02	0.00	0.00	0.04	0.00	0.01	0.01	0.00	0.01
Bacteria	*Desulfobulbus*	0.05	0.08	0.03	0.06	0.04	0.09	0.00	0.00	0.00	0.10	0.01	0.01
Bacteria	*Desulfurivibrio*	0.58	0.34	0.57	0.27	0.06	0.42	0.35	0.02	0.09	0.04	0.06	0.18
Bacteria	*Desulfofustis*	0.01	0.03	0.01	0.04	0.00	0.00	0.00	0.01	0.01	0.00	0.00	0.01
Bacteria	*Desulfomicrobium*	0.00	0.00	0.01	0.00	0.00	0.00	0.02	0.00	0.00	0.01	0.01	0.03
Bacteria	*Desulfovibrio*	2.12	2.56	2.56	2.37	0.67	0.47	3.15	0.15	0.82	2.79	1.86	0.83
Bacteria	*Desulfonauticus*	0.00	0.00	0.01	0.00	0.00	0.00	0.00	0.00	0.00	0.00	0.16	0.02
Bacteria	*Desulfarculus*	0.75	0.48	0.55	0.74	0.08	0.22	0.88	0.04	0.20	2.90	0.27	0.63
Bacteria	*Pelobacter*	0.47	0.24	0.30	0.36	0.22	0.15	0.11	0.00	0.03	0.04	0.04	0.06
Bacteria	*Desulfuromonas*	0.30	0.28	0.24	0.25	0.16	0.12	1.10	0.01	0.11	0.09	0.11	0.31
Bacteria	*Desulfoglaeba*	0.04	0.05	0.07	0.01	0.04	0.07	1.31	0.06	0.49	0.54	0.20	0.50
Bacteria	*Thermodesulforhabdus*	0.48	0.02	0.03	0.00	0.08	3.10	1.15	0.00	0.02	0.01	0.21	5.78
Bacteria	*Syntrophobacter*	0.02	0.05	0.04	0.06	0.00	0.00	0.03	0.01	0.01	0.40	0.02	0.11
Bacteria	*Syntrophorhabdus*	0.02	0.02	0.04	0.03	0.00	0.00	0.05	0.01	0.01	0.30	0.02	0.13
Bacteria	*Smithella*	0.26	0.14	0.21	0.10	0.00	0.07	1.23	0.02	0.15	0.07	0.13	0.03
Bacteria	*Roseovarius*	11.33	4.84	4.19	4.90	0.80	1.99	14.77	3.66	1.81	17.72	5.49	14.27
Bacteria	*Rubellimicrobium*	0.01	0.01	0.02	0.05	0.02	0.04	0.00	0.00	0.00	0.01	0.01	0.00
Bacteria	*Rhodovulum*	0.01	0.02	0.03	0.02	0.02	0.00	0.04	0.01	0.01	0.08	0.02	0.08
Bacteria	*Stappia*	1.00	0.91	0.89	1.20	0.27	0.26	0.09	0.09	0.11	0.51	0.12	0.56
Bacteria	*Paracoccus*	0.12	0.38	0.90	0.65	2.85	0.75	0.00	0.00	0.14	0.00	0.01	0.14
Bacteria	*Tistrella*	2.27	2.15	1.60	1.64	0.59	1.12	2.43	0.08	0.61	1.62	0.97	0.69
Bacteria	*Oceanibaculum*	0.21	0.34	0.33	0.32	0.06	0.13	0.45	0.14	1.23	0.84	1.95	0.38
Bacteria	*Thalassospira*	0.00	0.05	0.01	0.02	0.00	0.00	0.01	0.00	0.00	0.01	0.00	0.01
Bacteria	*Roseomonas*	0.01	0.00	0.01	0.00	0.00	0.07	0.01	0.00	0.00	0.00	0.01	0.04
Bacteria	*Methylobacterium*	0.04	0.04	0.05	0.10	0.10	0.18	0.03	0.00	0.00	0.07	0.03	0.01
Bacteria	*Bosea*	0.09	0.00	0.02	0.02	0.00	0.01	0.01	0.00	0.00	0.01	0.00	0.01
Bacteria	*Xanthobacter*	0.07	0.02	0.02	0.06	0.04	0.04	0.00	0.00	0.00	0.00	0.00	0.00
Bacteria	*Rhizobium*	0.01	0.00	0.00	0.00	0.02	0.00	0.01	0.01	0.01	0.00	0.00	0.02
Bacteria	*Novosphingobium*	0.00	0.00	0.01	0.00	0.04	0.12	0.00	0.00	0.00	0.00	0.01	0.01
Bacteria	*Sphingomonas*	0.01	0.00	0.03	0.08	0.16	0.26	0.00	0.00	0.00	0.07	0.01	0.11
Bacteria	*Sphingobium*	0.28	0.08	0.13	0.05	0.00	0.07	0.52	0.00	0.00	0.02	0.00	0.03
Bacteria	*Sphingopyxis*	0.01	0.00	0.00	0.01	0.00	0.08	0.00	0.00	0.00	0.01	0.00	0.00
Bacteria	*Caulobacter*	0.01	0.05	0.06	0.06	0.59	0.33	0.00	0.00	0.00	0.01	0.00	0.01
Bacteria	*Brevundimonas*	0.25	0.16	0.06	0.12	0.08	0.20	0.01	0.01	0.00	0.06	0.02	0.06
Bacteria	*Phenylobacterium*	0.10	0.06	0.07	0.05	0.14	0.07	0.34	0.01	0.02	0.25	0.03	0.14
Bacteria	*Hyphomonas*	0.03	0.01	0.14	0.06	0.02	0.03	0.15	0.02	0.07	0.11	0.12	0.09
Bacteria	*Achromobacter*	1.82	4.27	3.62	4.72	16.47	8.59	0.28	0.08	0.02	0.11	0.01	0.07

Bacteria	*Ralstonia*	0.01	0.03	0.01	0.01	0.14	0.82	0.00	0.00	0.00	0.10	0.07	0.61
Bacteria	*Pelomonas*	0.01	0.11	0.03	0.02	0.43	0.17	0.00	0.00	0.00	0.00	0.01	0.00
Bacteria	*Comamonas*	0.00	0.01	0.00	0.00	0.10	0.96	0.00	0.00	0.00	0.00	0.15	0.78
Bacteria	*Aquabacterium*	0.00	0.01	0.01	0.03	0.02	0.08	0.00	0.00	0.00	0.09	0.00	0.01
Bacteria	*Naxibacter*	0.01	0.01	0.01	0.02	0.02	0.09	0.01	0.00	0.00	0.04	0.00	0.00
Bacteria	*Janthinobacterium*	0.00	0.02	0.01	0.02	0.12	0.07	0.00	0.00	0.00	0.00	0.00	0.03
Bacteria	*Thiobacillus*	0.00	0.05	0.03	0.02	0.00	0.04	0.01	0.00	0.01	0.00	0.00	0.00
Bacteria	*Simplicispira*	0.00	0.00	0.05	0.03	0.00	0.00	0.00	0.00	0.00	0.01	0.00	0.00
Bacteria	*Acidovorax*	0.01	0.02	0.01	0.01	0.04	0.00	0.00	0.00	0.00	0.01	0.01	0.00
Bacteria	*Schlegelella*	0.00	0.01	0.00	0.01	0.00	0.01	0.00	0.00	0.00	0.00	0.00	0.00
Bacteria	*Tepidiphilus*	0.15	0.04	0.05	0.10	0.00	0.01	3.10	0.04	0.50	0.42	0.55	0.51
Bacteria	*Thauera*	0.02	0.18	0.12	0.11	0.00	0.04	0.00	0.03	0.01	0.06	0.01	0.03
Bacteria	*Sulfuritalea*	0.00	0.00	0.00	0.00	0.02	0.00	0.00	0.00	0.00	0.00	0.00	0.00
Bacteria	*Georgfuchsia*	0.00	0.00	0.00	0.00	0.00	0.04	0.00	0.00	0.00	0.00	0.00	0.00
Bacteria	*Dechloromonas*	0.01	0.00	0.00	0.01	0.00	0.03	0.01	0.00	0.01	0.01	0.00	0.01
Bacteria	*Azospira*	0.00	0.00	0.00	0.00	0.14	0.94	0.00	0.00	0.00	0.00	0.00	0.02
Bacteria	*Azoarcus*	0.00	0.00	0.00	0.01	0.00	0.00	0.00	0.00	0.01	0.01	0.00	0.01
Bacteria	*Methylophilus*	0.00	0.11	0.02	0.01	0.06	0.22	0.00	0.00	0.00	0.01	0.00	0.00
Bacteria	*Pseudomonas*	0.76	1.46	2.03	1.58	5.24	2.92	0.06	0.29	0.14	0.41	0.37	0.29
Bacteria	*Acinetobacter*	0.26	0.30	0.65	0.46	0.55	0.87	0.01	0.01	0.01	0.05	0.07	0.19
Bacteria	*Psychrobacter*	0.04	0.08	0.04	0.06	0.37	0.17	0.00	0.00	0.00	0.00	0.00	0.01
Bacteria	*Thioalkalimicrobium*	0.10	0.36	0.30	0.17	0.02	0.12	0.00	0.13	0.04	0.04	0.04	0.08
Bacteria	*Nitrincola*	0.86	1.65	2.83	1.46	0.41	1.25	0.14	0.69	0.20	0.10	0.11	0.23
Bacteria	*Thalassolituus*	0.46	0.41	0.49	0.14	0.10	0.18	0.05	0.21	0.04	0.02	0.07	0.08
Bacteria	*Halomonas*	0.17	0.18	0.29	0.35	0.31	0.28	0.01	0.01	0.02	0.32	0.60	0.12
Bacteria	*Alcanivorax*	1.29	0.61	0.40	0.25	0.10	0.22	0.16	0.01	0.04	0.09	0.07	0.14
Bacteria	*Legionella*	0.88	0.56	0.70	1.10	0.63	0.24	0.05	0.00	0.00	0.01	0.01	0.01
Bacteria	*Stenotrophomonas*	0.19	0.06	0.06	0.02	0.04	0.21	0.01	0.00	0.01	0.00	0.01	0.02
Bacteria	*Marinobacter*	0.40	0.33	0.67	1.01	0.24	0.25	0.05	2.99	0.37	0.82	1.51	0.51
Bacteria	*Marinobacterium*	1.16	2.39	2.94	2.12	0.53	1.15	0.35	10.07	2.01	2.24	1.81	1.49
Bacteria	*Alishewanella*	0.00	0.01	0.01	0.01	0.00	0.01	0.00	0.00	0.00	0.00	0.00	0.00
Bacteria	*Rheinheimera*	0.01	0.02	0.03	0.01	0.04	0.04	0.00	0.00	0.00	0.00	0.00	0.00
Bacteria	*Arcobacter*	0.06	0.04	0.08	0.07	0.00	0.01	0.01	0.08	0.01	0.04	0.09	0.02
Bacteria	*Sulfurimonas*	0.02	15.08	3.06	0.06	0.20	0.04	0.01	71.76	0.04	0.11	0.23	0.25
Bacteria	*Carnobacterium*	0.10	0.66	0.21	0.24	2.30	2.16	0.00	0.00	0.00	0.00	0.00	0.00
Bacteria	*Leuconostoc*	0.15	0.59	0.15	0.32	2.04	1.23	0.00	0.00	0.00	0.00	0.00	0.00
Bacteria	*Streptococcus*	0.02	0.04	0.04	0.02	0.08	0.28	0.00	0.00	0.00	0.01	0.00	0.03
Bacteria	*Solibacillus*	0.09	0.39	0.14	0.28	2.02	1.12	0.00	0.00	0.00	0.00	0.00	0.00
Bacteria	*Lysinibacillus*	0.10	0.25	0.04	0.17	1.41	0.77	0.00	0.00	0.00	0.00	0.00	0.00
Bacteria	*Bacillus*	0.34	1.65	0.90	1.59	9.19	5.58	0.00	0.00	0.00	0.01	0.00	0.01
Bacteria	*Staphylococcus*	0.03	0.00	0.00	0.00	0.16	0.11	0.00	0.00	0.00	0.01	0.00	0.00
Bacteria	*Paenibacillus*	0.06	0.19	0.28	0.25	1.53	1.28	0.00	0.00	0.00	0.00	0.00	0.00
Bacteria	*Saccharofermentans*	0.73	0.40	0.32	0.55	0.10	0.08	0.00	0.00	0.00	0.01	0.00	0.01
Bacteria	*Acetobacterium*	0.10	0.02	0.04	0.03	0.04	0.01	0.00	0.00	0.00	0.00	0.00	0.00

Bacteria	*Pelotomaculum*	0.01	0.06	0.04	0.06	0.00	0.00	0.01	0.00	0.00	0.02	0.01	0.01
Bacteria	*Desulfotomaculum*	0.00	0.00	0.00	0.00	0.00	0.38	0.00	0.00	0.00	0.00	0.00	0.39
Bacteria	*Desulfitobacterium*	0.10	0.02	0.02	0.02	0.00	0.00	0.04	0.00	0.00	0.01	0.00	0.00
Bacteria	*Proteiniclasticum*	0.05	0.05	0.17	0.25	0.10	0.09	0.00	0.00	0.00	0.00	0.00	0.00
Bacteria	*Syntrophorthermus*	0.01	0.02	0.03	0.01	0.00	0.01	0.01	0.00	0.00	0.00	0.00	0.03
Bacteria	*Syntrophomonas*	0.01	0.00	0.01	0.00	0.00	0.00	0.00	0.00	0.00	0.00	0.00	0.00
Bacteria	*Thermosyntropha*	0.01	0.00	0.01	0.01	0.00	0.01	0.00	0.00	0.00	0.00	0.00	0.14
Bacteria	*Anaerovorax*	0.51	0.38	0.26	0.33	0.02	0.08	0.00	0.00	0.00	0.00	0.00	0.00
Bacteria	*Dethiosulfatibacter*	1.09	0.82	0.78	0.82	0.22	0.40	0.00	0.00	0.01	0.03	0.01	0.01
Bacteria	*Thermacetogenium*	0.15	0.07	0.62	1.18	0.59	3.21	0.05	0.01	0.01	0.46	0.15	0.27
Bacteria	*Moorella*	0.00	0.02	0.00	0.00	0.00	0.09	0.00	0.00	0.00	0.00	0.00	0.03
Bacteria	*Sphaerochaeta*	1.97	1.29	1.31	0.96	0.14	0.57	1.58	0.05	0.46	0.15	0.09	0.14
Bacteria	*Spirochaeta*	0.12	0.11	0.16	0.26	0.10	0.37	0.34	0.02	0.09	0.04	0.01	0.19
Bacteria	*Proteiniphilum*	0.26	0.12	0.21	0.26	0.00	0.08	0.03	0.01	0.04	0.49	0.06	0.12
Bacteria	*Paludibacter*	0.14	0.05	0.08	0.09	0.00	0.01	0.01	0.00	0.01	0.01	0.00	0.01
Bacteria	*Hymenobacter*	0.01	0.00	0.03	0.00	0.00	0.03	0.00	0.00	0.00	0.00	0.00	0.01
Bacteria	*Fontibacter*	0.06	0.03	0.07	0.16	0.08	0.00	0.02	0.00	0.04	0.25	0.02	0.04
Bacteria	*Flavobacterium*	0.01	0.03	0.03	0.02	0.04	0.16	0.00	0.00	0.00	0.00	0.00	0.03
Bacteria	*Ignavibacterium*	1.63	1.47	1.33	1.39	0.43	0.80	1.84	0.11	1.28	0.41	0.23	0.83
Bacteria	*Thermodesulfobacterium*	0.06	0.05	0.07	0.08	0.24	0.08	0.45	0.06	0.09	0.95	1.15	0.33
Bacteria	*Caldimicrobium*	0.00	0.00	0.00	0.00	0.00	0.03	0.01	0.00	0.00	0.00	0.03	0.09
Bacteria	*Deferribacter*	0.19	0.14	0.14	0.17	0.02	0.05	0.76	0.01	0.12	0.20	0.05	0.12
Bacteria	*Geovibrio*	0.04	0.00	0.06	0.06	0.02	0.00	0.06	0.00	0.02	0.02	0.02	0.06
Bacteria	*Geotoga*	1.31	1.88	1.32	1.70	0.20	0.53	5.40	0.30	4.19	0.64	0.69	0.64
Bacteria	*Thermotoga*	0.00	0.01	0.01	0.11	0.10	0.41	0.02	0.00	0.01	0.11	0.08	0.21
Bacteria	*Thermosipho*	0.01	0.03	0.00	0.02	0.00	0.01	0.01	0.00	0.01	0.01	0.00	0.03
Bacteria	*Kosmotoga*	0.06	0.07	0.06	0.03	0.00	0.00	0.06	0.00	0.01	0.03	0.04	0.00
Bacteria	*Thermovirga*	0.06	0.03	0.03	0.02	0.02	0.03	0.02	0.01	0.00	0.04	0.82	0.41
Bacteria	*Aminobacterium*	0.03	0.02	0.02	0.02	0.00	0.00	0.00	0.01	0.00	0.02	0.02	0.01
Bacteria	*Arthrobacter*	0.43	1.41	0.43	0.89	7.54	3.76	0.00	0.00	0.00	0.00	0.01	0.00
Bacteria	*Microbacterium*	0.06	0.06	0.02	0.03	0.00	0.04	0.00	0.00	0.00	0.00	0.00	0.00
Bacteria	*Propionibacterium*	0.00	0.03	0.02	0.04	0.02	0.25	0.00	0.00	0.00	0.01	0.00	0.01
Bacteria	*Thermodesulfovibrio*	0.00	0.03	0.02	0.02	0.00	0.04	0.00	0.00	0.01	0.01	0.00	0.19
Archaea	*Thermococcus*	1.47	0.97	1.03	1.18	2.08	1.60	0.18	0.22	0.15	0.03	0.04	0.27
Archaea	*Methanobrevibacter*	0.06	0.02	0.00	0.00	0.55	0.04	0.00	0.00	0.00	0.00	0.00	0.00
Archaea	*Methanothermobacter*	9.27	7.81	3.56	1.28	0.76	1.94	0.03	0.01	0.01	0.00	0.00	0.01
Archaea	*Methanobacterium*	0.20	0.30	0.07	0.03	0.01	0.00	0.01	0.01	0.01	0.05	0.00	0.01
Archaea	*Methanosphaera*	0.00	0.00	0.00	0.00	0.00	0.00	0.00	0.00	0.00	0.04	0.00	0.00
Archaea	*Methanothermus*	0.00	0.00	0.02	0.17	0.98	0.00	0.00	0.00	0.00	0.00	0.00	0.00
Archaea	*Methermicoccus*	0.00	0.06	0.04	0.00	0.34	0.38	0.00	0.03	0.10	0.01	0.65	1.23
Archaea	*Methanolobus*	46.58	46.38	33.42	8.55	2.67	3.67	3.83	60.57	5.23	0.09	0.26	6.71
Archaea	*Methanosaeta*	0.60	9.28	9.15	0.63	0.25	0.35	0.01	1.22	0.43	0.21	0.91	0.98
Archaea	*Methanosarcina*	0.02	0.00	0.01	0.00	0.00	0.00	0.00	0.00	0.00	0.00	0.00	0.00
Archaea	*Methanococcoides*	0.57	0.25	0.11	0.21	0.00	0.00	0.01	0.14	0.01	0.00	0.00	0.05

Archaea	*Methanohalophilus*	0.00	0.03	0.00	0.02	0.00	0.00	0.00	0.00	0.00	0.00	0.00	0.00
Archaea	*Methanofollis*	0.00	0.09	0.03	0.02	0.00	0.01	0.00	0.03	0.05	0.01	0.00	0.00
Archaea	*Methanolinea*	0.00	0.00	0.00	0.00	0.00	0.00	0.00	0.00	0.00	0.00	0.00	0.01
Archaea	*Methanoculleus*	0.00	0.02	0.01	0.00	0.00	0.00	0.03	0.01	0.01	0.00	0.00	0.01
Archaea	*Methanocalculus*	0.85	0.04	0.06	0.02	0.00	0.02	0.62	0.06	0.07	0.00	0.00	0.29
Archaea	*Archaeoglobus*	26.09	27.18	46.30	84.10	84.08	73.86	91.48	36.86	93.11	99.39	97.89	89.18
Archaea	*Candidatus Nitrosopumilus*	0.00	0.00	0.00	0.00	0.00	0.07	0.00	0.00	0.00	0.00	0.00	0.00
Archaea	*Nanoarchaeum*	0.16	0.05	0.15	0.23	0.80	0.26	0.02	0.00	0.02	0.00	0.02	0.03
Archaea	*Candidatus Caldiarchaeum*	0.01	0.00	0.02	0.00	0.00	0.19	0.00	0.00	0.00	0.00	0.00	0.06
Archaea	*Ignisphaera*	0.00	0.00	0.00	0.00	0.11	3.08	0.00	0.00	0.00	0.00	0.00	0.03
Archaea	*Thermofilum*	0.01	0.02	0.02	0.11	0.07	2.03	0.00	0.00	0.00	0.00	0.00	0.01
Archaea	*Terrestrial Miscellaneous Gp(TMCG)*	0.11	0.10	0.06	0.02	0.00	0.07	0.02	0.08	0.03	0.00	0.00	0.26
Archaea	*Woesearchaeota (DHVEG-6)*	3.87	1.98	1.17	0.20	0.88	0.14	0.19	0.13	0.01	0.00	0.00	0.00
Archaea	*Marine Group I*	0.00	0.00	0.00	0.00	0.00	0.14	0.00	0.00	0.00	0.00	0.00	0.00
Archaea	*Soil Crenarchaeotic Group(SCG)*	0.00	0.00	0.00	0.00	0.13	0.00	0.00	0.00	0.00	0.00	0.01	0.00
Archaea	*terrestrial group*	0.00	0.00	0.00	0.00	0.72	0.01	0.00	0.00	0.00	0.00	0.00	0.00
Archaea	*Marine Group II*	0.00	0.00	0.00	0.00	0.00	0.13	0.00	0.00	0.00	0.00	0.00	0.00
Archaea	*Marine Group III*	0.00	0.00	0.00	0.00	0.00	0.04	0.00	0.00	0.00	0.00	0.00	0.00
Archaea	*South African Goldmine Gp(SAGMEG)*	0.01	0.08	0.02	0.00	0.00	0.04	0.00	0.00	0.00	0.00	0.00	0.00
Archaea	*Marine Benthic Group D and DHVEG-1*	0.00	0.54	0.07	0.00	0.00	0.12	0.00	0.01	0.03	0.00	0.00	0.09
Archaea	*Kazan-3A-21*	6.67	1.92	1.38	0.35	0.62	0.07	2.97	0.04	0.07	0.10	0.01	0.03
Archaea	*Methanocorpusculum*	0.00	0.00	0.00	0.00	0.00	0.00	0.00	0.00	0.00	0.00	0.01	0.00

Table S4. Spearman's rank correlations (r_s) between genomic taxa and environmental factors. Significant correlations ($|rs| > 0.75$, $P < 0.05$) displayed.[a]

	Depth	pH	CR[b]	TDS	Mg^{2+}	Ca^{2+}	SO_4^{2-}	CO_3^{2-}	Cl^-	NO_3^-	S^{2-}	$S_2O_3^{2-}$
**Bacteria**												
Sphingobacteriia	0.88	ns	ns	ns	0.97	0.97	ns	-0.88	0.91	ns	ns	ns
Spirochaetia	-0.94	ns	ns	ns	ns	0.94	ns	1.00	ns	ns	ns	ns
Thermodesulfobacteria	0.94	-0.83	ns	ns	ns	ns	ns	-0.89	ns	ns	ns	ns
Ignavibacteria	-0.89	ns	ns	ns	ns	-0.83	ns	0.94	ns	ns	ns	ns
Gammaproteobacteria	ns	ns	-0.89	ns	ns	ns	ns	ns	ns	ns	0.89	0.94
Deltaproteobacteria	ns	ns	ns	ns	ns	ns	ns	0.83	ns	ns	ns	ns
Bacteroidia	ns	ns	-0.89	ns	ns	ns	ns	ns	ns	ns	0.89	0.94
Chlorobia	ns	ns	-0.94	ns	ns	ns	ns	ns	ns	ns	0.83	0.89
Thermotogae	ns	ns	-0.89	ns	ns	ns	ns	ns	ns	ns	0.89	0.83
Betaproteobacteria	ns	ns	ns	0.83	ns	0.89	ns	-0.94	ns	ns	ns	ns
Synergistia	ns	ns	ns	ns	ns	ns	ns	ns	ns	-0.89	ns	ns
Bacilli	ns	ns	ns	ns	ns	ns	ns	-0.83	ns	ns	ns	ns
Nitrospira	ns	ns	ns	ns	ns	ns	ns	-0.93	ns	ns	ns	ns
Deferribacteres	ns	ns	ns	ns	ns	ns	ns	ns	ns	ns	0.88	ns
Anaerolineae	ns	ns	ns	ns	ns	ns	ns	ns	ns	ns	ns	0.83
Actinobacteria	ns	ns	ns	ns	ns	ns	ns	ns	ns	ns	ns	-0.84
Clostrida	ns	ns	ns	ns	ns	ns	ns	ns	ns	ns	ns	ns
Alphaproteobacteria	ns	ns	ns	ns	ns	ns	ns	ns	ns	ns	ns	ns
**Archaea**												
Thermococci	0.83	ns	ns	ns	ns	ns	ns	ns	ns	ns	ns	ns
Methanobacteria	ns	0.94	ns	ns	ns	-0.83	ns	ns	-0.83	ns	ns	ns
Methanomicrobia	-1.00	ns	ns	ns	-0.83	-0.89	ns	0.94	ns	ns	ns	ns
Archaeoglobi	0.89	ns	ns	0.83	0.89	1.00	ns	-0.94	0.89	ns	ns	ns
Thermoplasmata	ns	0.94	ns	ns	ns	-0.83	ns	ns	-0.83	ns	ns	ns
Woesearchaeota	ns	0.89	ns	ns	ns	ns	ns	-0.90	ns	ns	ns	ns
Methanococci	ns	ns	-0.88	ns	ns	ns	ns	ns	ns	ns	ns	ns
Crenarchaeota	ns	ns	0.90	ns	ns	ns	ns	ns	ns	ns	ns	ns

[a]Non-significant (ns) values not displayed on the table.

[b]CR represents corrosion rate.

Table S5. Spearman's rank correlations (r_s) between active taxa and environmental factors. Significant correlations (|rs| >0.75, P < 0.05) displayed.

	Depth	pH	CR	TDS	Mg^{2+}	Ca^{2+}	SO_4^{2-}	CO_3^{2-}	Cl-	NO_3^-	Na^+	Formate	Acetate
**Bacteria**													
Sphingobacteriia	ns	-0.85	ns	ns	ns	ns	ns	ns	ns	ns	ns	ns	ns
Spirochaetia	ns	ns	ns	-0.83	ns	ns	ns	ns	ns	ns	ns	ns	ns
Thermodesulfobacteria	ns	-0.89	ns	ns	ns	ns	0.89	ns	ns	ns	ns	ns	ns
Ignavibacteria	ns	ns	ns	-0.83	ns	ns	ns	ns	ns	ns	ns	ns	ns
Gammaproteobacteria	ns	ns	ns	ns	0.83	ns	ns	ns	ns	ns	ns	ns	ns
Deltaproteobacteria	ns	ns	ns	ns	ns	ns	ns	ns	ns	ns	ns	ns	ns
Bacteroidia	ns	-1.00	ns	ns	ns	ns	ns	ns	ns	ns	ns	ns	ns
Chlorobia	ns	ns	ns	ns	ns	ns	ns	ns	ns	ns	ns	ns	ns
Thermotogae	ns	ns	ns	ns	ns	ns	ns	ns	ns	ns	ns	ns	ns
Betaproteobacteria	ns	ns	ns	ns	ns	ns	ns	ns	ns	-0.89	ns	ns	ns
Synergistia	ns	ns	ns	ns	ns	ns	ns	ns	ns	ns	ns	ns	ns
Bacilli	ns	ns	ns	ns	ns	ns	ns	ns	ns	ns	ns	ns	ns
Nitrospira	ns	ns	ns	ns	ns	ns	ns	ns	ns	ns	ns	ns	ns
Deferribacteres	ns	ns	ns	ns	ns	ns	ns	ns	ns	-0.83	ns	ns	ns
Anaerolineae	ns	ns	ns	ns	ns	ns	ns	ns	ns	ns	ns	ns	ns
Actinobacteria	ns	-0.93	ns	ns	ns	ns	ns	ns	ns	ns	ns	ns	ns
Clostrida	ns	-0.89	ns	ns	ns	ns	ns	ns	ns	ns	ns	ns	ns
Alphaproteobacteria	ns	ns	ns	ns	ns	ns	ns	ns	ns	ns	ns	ns	ns
**Archaea**													
Thermococci	ns	ns	ns	ns	ns	ns	ns	ns	ns	ns	-1	-1	-0.89
Methanobacteria	ns	ns	ns	ns	ns	ns	ns	ns	ns	ns	ns	ns	ns
Methanomicrobia	ns	ns	ns	ns	ns	ns	-0.89	ns	ns	ns	ns	-0.89	-0.89
Archaeoglobi	ns	ns	ns	ns	0.88	ns	0.88	ns	ns	ns	0.91	0.91	0.97
Thermoplasmata	ns	ns	ns	ns	ns	ns	ns	ns	ns	-0.89	ns	ns	ns
Woesearchaeota	ns	ns	ns	ns	ns	ns	ns	ns	ns	ns	ns	ns	ns
Methanococci	ns	ns	ns	ns	ns	ns	ns	ns	ns	ns	ns	ns	ns
Crenarchaeota	ns	ns	ns	ns	ns	ns	ns	ns	ns	ns	-0.97	-0.97	-0.91

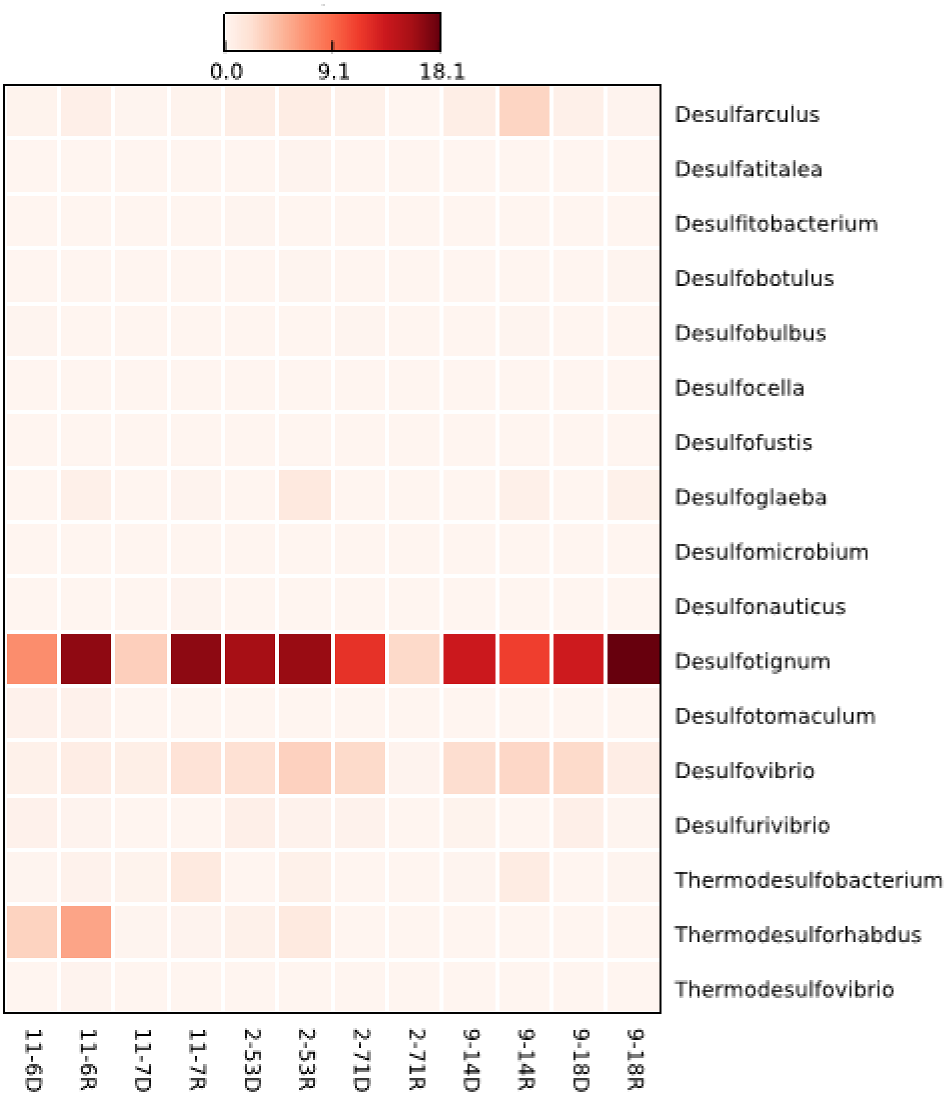

Figure S1 Heatmap showing distribution of SRM inferred from 16S rRNA gene (DNA-based) and 16S rRNA (RNA-based) high-throughput sequencing. Colour bar represent the relative percentage.

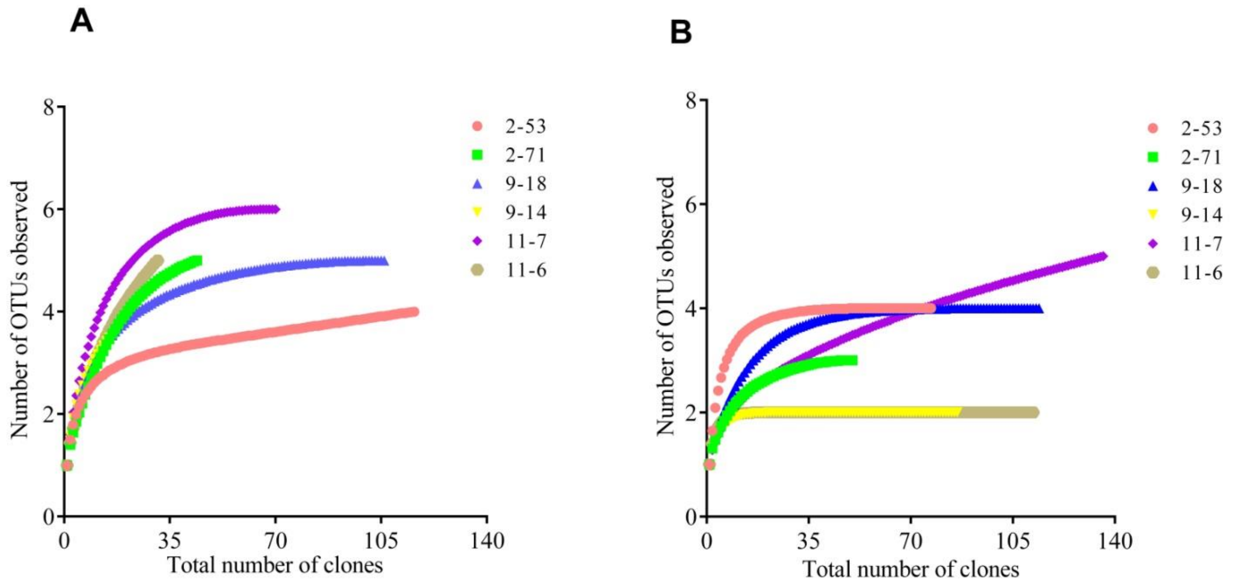

Figure S2 Rarefaction curves displaying observed OTU richness versus the number of *aprA* (A) and *dsrA* (B) gene clones sequenced from each of production water of six different oil well samples.
